# Radiotherapy plus a self-gelation powder encapsulating tRF5-GlyGCC inhibitor potentiates natural kill cell immunity to prevent hepatocellular carcinoma recurrence

**DOI:** 10.1186/s12951-025-03133-3

**Published:** 2025-02-10

**Authors:** Yihang Gong, Fanxin Zeng, Feng Zhang, Xiaoquan Liu, Zhongheng Li, Wenjie Chen, Haipeng Liu, Xin Li, Yusheng Cheng, Jian Zhang, Yeqian Feng, Tiangen Wu, Wence Zhou, Tong Zhang

**Affiliations:** 1 Department of Hepatic Surgery and Liver Transplantation Center, The Third Affiliated Hospital of Sun Yat-Sen University, Organ Transplantation Institute, Organ Transplantation Research Center of Guangdong Province, Guangdong Province Engineering Laboratory for Transplantation Medicine, Sun Yat-Sen University, Guangzhou, 510630 China; 2 Biotherapy Centre & Cell-Gene Therapy Translational Medicine Research Centre, The Third Affiliated Hospital, Sun Yat-Sen University, Guangzhou, 510630 China; 3 Department of Infectious Diseases, Third Affiliated Hospital of Sun Yat-Sen University, Guangzhou, 510630 Guangdong China; 4 Department of General Surgery, Lanzhou University Second Hospital, Lanzhou, 730000 China; 5 Department of Oncology, The Second Xiangya Hospital, Central South University, Changsha, 410011 Hunan China; 6 Department of Hepatobiliary & Pancreatic Surgery, Zhongnan Hospital of Wuhan University, Wuhan, 430071 Hubei People’s Republic of China; 7 Organ Transplantation Clinical Medical Center of Xiamen University, Department of General Surgery, Xiang’an Hospital of Xiamen University, School of Medicine, Xiamen University, Xiamen, 361102 China; 8 Organ Transplantation Institute of Xiamen University, Xiamen Human Organ Transplantation Quality Control Center, Xiamen Key Laboratory of Regeneration Medicine, Fujian Provincial Key Laboratory of Organ and Tissue Regeneration, School of Medicine, Xiamen University, Xiamen, 361102 China

**Keywords:** Hepatocellular carcinoma, TRNA-derived fragments, NK cell immunity, Radiotherapy, Nanocomposite hydrogel

## Abstract

Hepatocellular carcinoma (HCC) recurrence postresection represents a thorny problem in clinical practice, of which impaired natural killer (NK) cell cytotoxicity represents one of crucial causes. Apart from recurrence, hepatectomy-induced abdominal adhesion also poses huge clinical challenges such as abdominal pain, intestinal obstruction, and perforation. Evidence demonstrates that radiotherapy can upregulate NK group 2D ligand expression on tumor cells to enhance NK cell cytotoxicity, indicating its great potential of curbing HCC recurrence. Nevertheless, radiotherapy has also been disclosed to incur suppression on NK antitumor cell immunity. Herein, we reveal that glycocholic acid (GCA)/tRNA-derived fragment 5 (tRF5)-GlyGCC signaling axis is activated in mouse HCC model after radiotherapy, which dampens NK cell antitumor immunity to limit therapeutic efficacy. Mechanistically, tRF5-GlyGCC can interact with KDM6B to epigenetically upregulate Runx2 and then transcriptionally activate ITGBL1 and S100A9 expression in HCC cells, which further reduces NK cell cytotoxicity directly and attracts myeloid-derived suppressor cell (MDSC) to inhibit NK cell function indirectly, respectively. Therefore, radiotherapy plus targeting tRF5-GlyGCC may be an optimized postoperative adjuvant therapy against HCC recurrence. Then, a nanocomposite powder is designed for liver-localized delivery of tRF5-GlyGCC inhibitor. After sprayed to liver resection margin of mouse HCC model, this powder can rapidly form an in-situ Janus-adhesive hydrogel, which allows for sustained delivery of tRF5-GlyGCC inhibitor. Importantly, it can synergize with radiotherapy to potentiate NK cell antitumor immunity and prevent HCC recurrence postresection. Moreover, its application to surgical bed also effectively mitigates abdominal adhesion in a rat hepatectomy model. Altogether, our work develops a tRF5-GlyGCC-targeting nanocomposite power for sensitizing radiotherapy to thwart HCC recurrence and preventing abdominal adhesion.

**Graphical Abstract:**

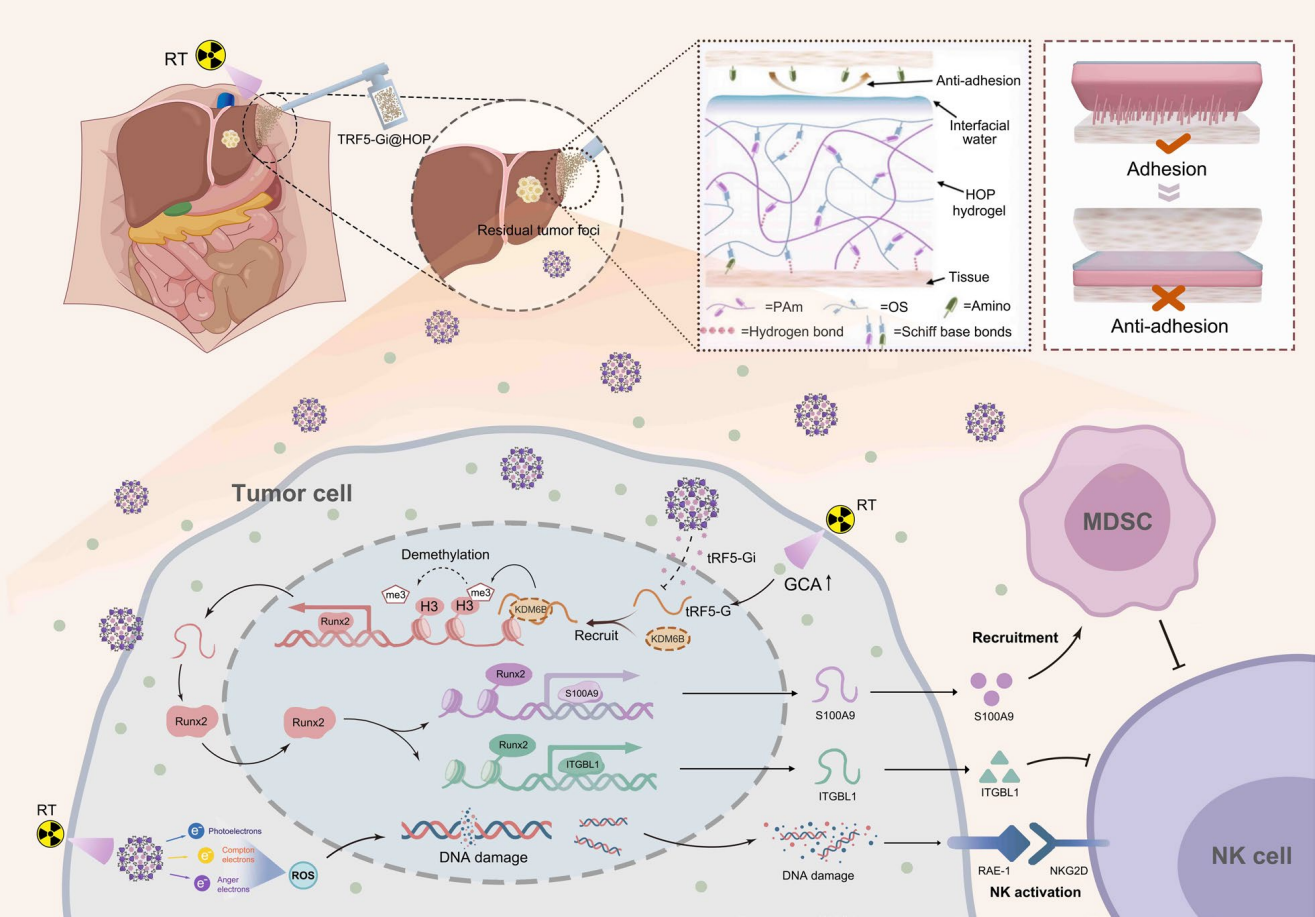

**Supplementary Information:**

The online version contains supplementary material available at 10.1186/s12951-025-03133-3.

## Introduction

Hepatocellular carcinoma (HCC) ranks as the fifth most prevalent form of human malignancy globally and the second leading cause of malignancy-related mortality [1, 2]. To date, liver resection is the mainstay of curative treatment for patients with HCC. The basic principle of surgical resection for HCC is to completely remove tumor masses, ensure a surgical margin of 1–2 cm from the lesions, and preserve normal hepatic tissue to reduce post-surgical mortality and complications [3]. However, tumor masses are close to major vessels in some patients with HCC; therefore, for surgeons to obtain a resection margin of less than 1 cm is difficult. In this scenario, HCC recurrence reaches 70% within two years postoperatively due to the presence of tumor foci, which severely shortens patient survival time [4]. In addition to HCC recurrence, hepatectomy-induced intra-abdominal adhesions pose significant clinical challenges, such as abdominal pain, intestinal obstruction, and even perforation [5]. In particular, some patients require repeated hepatectomy or liver transplantation due to HCC recurrence following the initial hepatectomy. However, the existence of perihepatic adhesions resulting from a previous hepatectomy significantly increases technical difficulties and hampers the feasibility of repeated surgical therapy. Accordingly, an urgent need exists to develop effective treatment strategies to combat postsurgical HCC recurrence and abdominal adhesions.

Natural killer (NK) cells are crucial components of the hepatic innate immune system, comprising 30%–50% of intrahepatic lymphocytes [6]. Moreover, NK cells can selectively recognize and kill tumor cells without pre-exposure to tumor-specific antigens, and this process does not depend on major histocompatibility complexes. Thus, NK cells play an essential role in clearing residual tumor foci around the surgical margin to prevent HCC recurrence post-resection [7]. However, NK cell cytotoxicity is severely impaired in patients undergoing hepatectomy for HCC, which is strongly associated with HCC recurrence post-resection and poor prognosis [8]. Solid evidence has demonstrated that NK group 2D (NKG2D) ligands, such as MICA/MICB in human HCC cells and RAE-1 in murine HCC cells, represent one of the determinant factors of NK cell cytotoxicity through interaction with the NKG2D receptor on NK cells [9, 10]. In recent years, radiotherapy (RT) has evolved into a pivotal component of the comprehensive treatment strategies for HCC. Notably, a growing number of studies have revealed that RT can upregulate NKG2D ligands to enhance NK cell cytotoxicity in tumor cells, apart from directly killing tumor cells [11, 12]. Moreover, clinical studies have shown that postoperative adjuvant RT can reduce tumor recurrence in patients receiving narrow-margin liver resection for centrally located HCC neighboring the major vessels [13, 14]. These findings imply that RT may be a promising strategy for reinstating NK cell cytotoxicity to prevent postsurgical recurrence of HCC. However, RT also suppresses the antitumor immunity of NK cells [15, 16]. Therefore, RT combined with strategies that relieve RT-induced immunosuppression may be an optimal therapeutic modality to combat postoperative HCC recurrence.

Bile acids (BAs) are metabolites produced in the liver and synthesized from cholesterol under the control of specific enzymes [17]. The aberrant metabolism of BAs has an intimate association with HCC [18, 19]. Mounting evidence suggests that BAs are involved in the regulation of tumor immunity in HCC. Our previous study demonstrated that norcholic acid increases PD-L1 levels in tumor cells to facilitate HCC cells in evading CD8^+^ T cell-mediated immune attack [20]. In addition, Gou et al. reported that obeticholic acid significantly enhanced antitumor immunity by increasing CXCR6^+^ natural killer T cell accumulation in HCC [21]. tRNA-derived fragments (tRFs) are a novel class of small non-coding RNAs produced by the specific cleavage of mature or precursor transfer RNAs, which have been shown to modulate various biological molecular processes such as oncogenic transformation, gene silencing, RNA processing, and protein biosynthesis [22]. In addition, several tRFs have been proven to be associated with tumor growth, metastasis, and immune evasion [23]. Herein, we for the first time reveal that the glycocholic acid (GCA)/tRNA-derived fragment 5 (tRF5)-GlyGCC signaling axis is activated in a mouse HCC model after radiotherapy, which dampens NK cell antitumor immunity and limits therapeutic efficacy. Mechanistically, tRF5-GlyGCC can interact with KDM6B to epigenetically upregulate Runx2 and transcriptionally activate ITGBL1 and S100A9 expression in HCC cells, which further reduces NK cell cytotoxicity directly and attracts myeloid-derived suppressor cells (MDSC) to inhibit NK cell function indirectly, respectively [24, 25]. Therefore, radiotherapy combined with targeting of tRF5-GlyGCC may be an optimal postoperative adjuvant therapy for HCC recurrence.

**Fig. 1:**
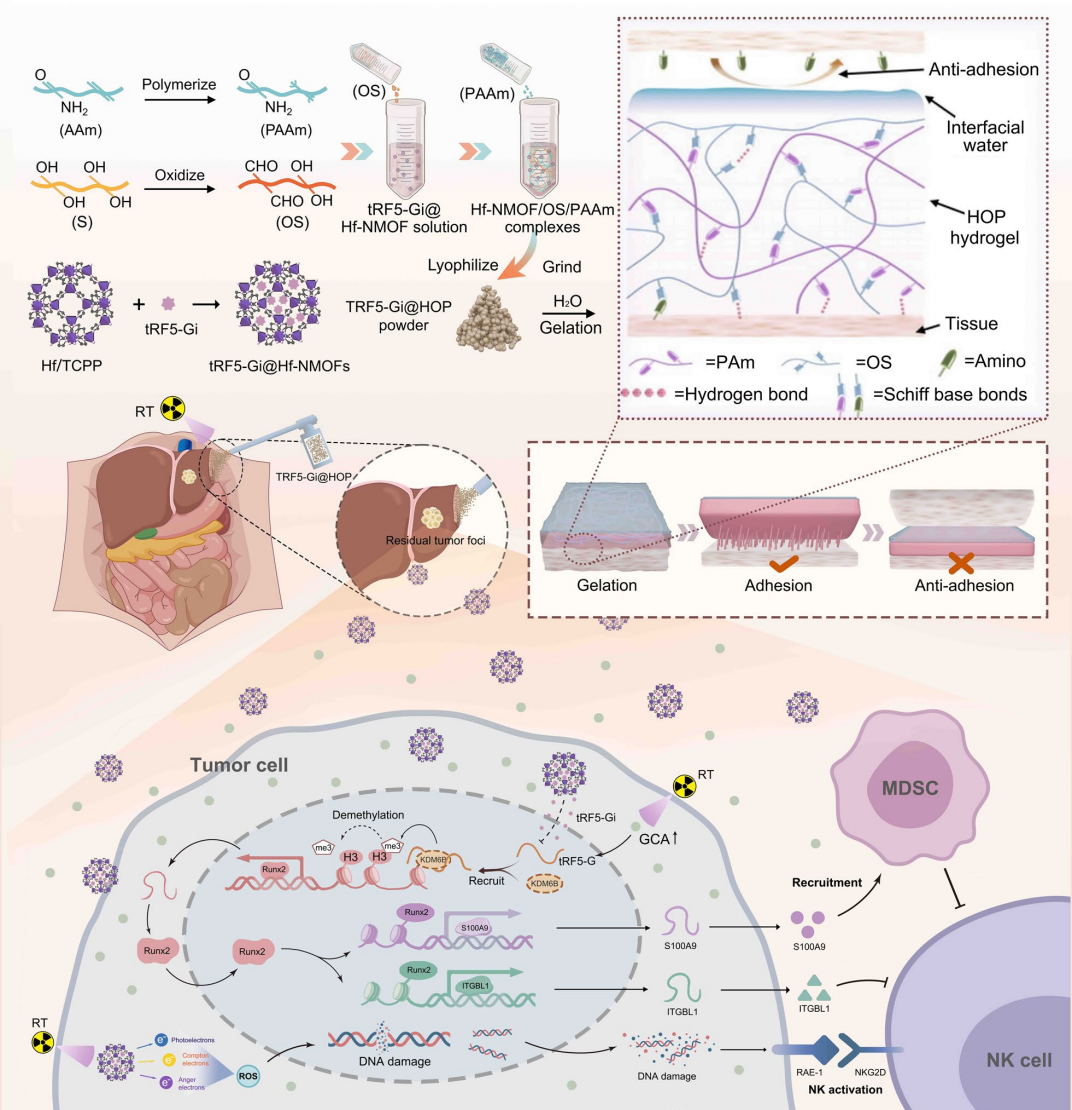
Radiotherapy plus a self-gelation powder encapsulating tRF5-GlyGCC inhibitor potentiates natural kill cell immunity to prevent hepatocellular carcinoma recurrence. The oxidized starch (OS), polyacrylamide (PAAm) hydrogel, and radiosensitized Hf^4+^/TCPP nanoscale metal organic framework (Hf/TCPP NMOFs) containing tRF5-GlyGCC inhibitor (tRF5-Gi) are first prepared, and then they are mixed and ground to generate a nanocomposite powder, termed as tRF5-Gi@HOP powder. After sprayed to surgical margin of mouse HCC model, this powder rapidly forms an in-situ hydrogel. This hydrogel can sustain a liver-localized delivery of tRF5-Gi and thereby synergize with RT to prevent HCC recurrence postresection via potentiating NK cell immunity. Additionally, tRF5-Gi@HOP powder can effectively mitigate abdominal adhesion in a rat hepatectomy model

To target tRF5-GlyGCC in HCC cells, we synthesized the tRF5-GlyGCC inhibitor (tRF5-Gi), a kind of single stranded nucleotide. However, the naked single stranded nucleotide against tRF5-GlyGCC may show low efficiency in inhibiting tRF5-GlyGCC due to its rapid degradation by nucleases in the blood and undergo rapid renal clearance in vivo, which is similar to naked small interfering RNA (siRNA) [26]. Therefore, it is imperative to develop a novel delivery platform of tRF5-GlyGCC inhibitor for improving its therapeutic efficacy. During the past decades, the polymer nanocomposite hydrogels used as novel delivery platforms of various drugs including siRNA have aroused accumulating interest in the field of cancer therapy [27], in that they can fulfil the controlled local drug delivery with high bioavailability thereby improving therapeutic effects. Nevertheless, the previously designed hydrogels are in liquid state or liquid-hydrogel transition state, so they show a poor performance in absorbing interfacial water when applied onto the surgical bed. This drawback substantially weakens the cross-linking between the functional groups in the hydrogels and those groups in the wet tissues, which may accelerate the hydrogel detachment from the surgical bed and limit its antitumor efficacy. To resolve this issue, herein we design a novel nanocomposite powder enabling liver-localized delivery of tRF5-Gi to formulate a radiotherapy-based combinatorial adjuvant treatment against HCC recurrence (Fig. 1). Briefly, Oxidized starch (OS), polyacrylamide (PAAm) hydrogel, and radiosensitized hafnium ion (Hf4 +)/tetrakis (4-carboxyphenyl) porphyrin (TCPP) nanoscale metal–organic framework (Hf/TCPP NMOFs) loaded with tRF5-Gi are sequentially prepared, mixed, and ground to generate a nanocomposite powder, termed as tRF5-Gi@HOP powder. Following spraying onto the surgical margin of the mouse HCC model, this powder can rapidly form an adhesive pressure-resistant hydrogel in situ after deposited to liver resection margin, fulfill sustained the liver-localized delivery of tRF5-Gi, and sensitize HCC cells to low-dose X-ray radiation. Moreover, this powder can synergize with RT to prevent HCC recurrence post-resection by potentiating NK cell antitumor immunity. The postoperative adhesion is another common morbidity in abdominal surgery, which may lead to lifelong risks, such as chronic pain, intestinal obstruction, and even intestinal perforation. Intriguingly, in this study we also observed that the application of tRF5-Gi@HOP powder to the surgical bed can effectively mitigate abdominal adhesions in a rat hepatectomy model, which may be due to the Janus-adhesive properties of its derived hydrogel. Taken together, our work develops a tRF5-GlyGCC-targeting nanocomposite for sensitizing radiotherapy to thwart HCC recurrence and preventing abdominal adhesions.

## Materials and methods

### Cells and animals

Huh-7 cells and Hepa1-6 cells were bought from the American Typical Culture Collection (ATCC), which were cultivated in Dulbecco’s Modified Eagle’s Medium containing 10% fetal bovine serum (FBS) plus 0.1% penicillin–streptomycin. The mouse NK cells isolation was fulfilled using a Mojosort Mouse NK Cell Isolation Kit (Biolegend, Cat#480,049) and kept in medium with 10% FBS and IL-15 (1 ng/ml, Peprotech). Male mice (7–8 weeks, BALB/c) and SD rats (male, 250–300 g) were procured from SLAC Laboratory Animal Co. Ltd (Shanghai, China) and Chinese Academy of Sciences (Shanghai, China), respectively. The animal experiments were conducted in compliance with the ARRIVE guidelines and the U.K. Animals (Scientific Procedures) Act, 1986.

### The analysis of bile acid profiles

The ultra-performance liquid chromatography/tandem mass spectrometry was adopted for analyzing the bile acid profiles in mouse HCC tissues as we described previously [20].

### Lactate dehydrogenase release assay

NK cells were first cultivated alone and then underwent co-incubation with Hepa1-6 cells at several NK-to- Hepa1-6 ratios, and this co-cultivation persisted for six hours. After co-cultivation, we centrifuged the culture plates, and subsequently gathered the supernatants meticulously without disturbing the cell pellets. At last, we measured LDH activity in the supernatants through utilizing LDH release assay kit (Abcam, UK).

### Calcein AM staining assay

The calcein AM assay was carried out through utilizing the calcein AM staining kit in compliance with the factory's guidelines (CA1630, Solarbio, China). In short, Hepa1-6 cells were implanted into a 96-well plate pre-covered using poly-Lornithine. The culture medium with 1 μM calcein AM was dropped into the plates and subjected to incubation for 20 min. Following these steps, the murine NK cells were added to the plates according to the specified proportion and were co-incubated with HCC cells for a duration of 2 h. After co-cultivation, we washed cells were with PBS, and at last observed cells using fluorescence microscopy.

### ELISA and WB analysis

The concentrations of S100A8, S100A9, IL-6, and ITGBL1 in the supernatants of tumor cells or HCC tissues were measured through Enzyme-linked immuno sorbent assay (ELISA) following the instructions provided by the manufacturer. The ELISA kits for S100A8, S100A9, and IL-6 were purchased from RD systems, and the ITGBL1 ELISA kit was purchased from BYabscience. Western blot (WB) analysis was performed in compliance with our previously published study [9]. The primary antibodies against KDM6B, GAPDH, Histone H3, and Alpha-tublin were purchased from Abcam company.

### tRFs and mRNA sequencing

PANDORA-seq was performed to analyze tRF expression profiles based on the protocol reported by Shi et al. [28]. The total RNA was first extracted from control and GCA-knockdown Hepa1-6 cells using Trizol reagent (390,206, Life Technology, USA). LC-Bio Technologies (Hangzhou) Co.,Ltd. Provided mRNA sequencing service. The significantly expressed mRNAs were defined as those with fold alteration < 0.5 or fold alteration above 2 through utilizing parametric F-test comparing nested linear models (p value < 0.05) combined with R package edgeR (https://bioconductor.org/packages/release/bioc/html/edgeR. html).

### Quantitative real-time PCR (qRT-PCR)

Total RNA isolation was first implemented with the TRIzol reagents (Life Technologies, Shanghai China) in compliance with the producer’s guidance. Then, the Takara cDNA kit (Takara Bio, Beijing, China) was used for the reverse transcription of 1 mg total RNA into cDNA. The transcript levels of specific genes or tRFs were amplified with the Takara qPCR kit, and their expression levels were normalized to GAPDH and U6, respectively. The relevant primers were bought from MIAOLING BIOLOGY (Wuhan, China), the sequences of which were shown in Table S1.

### Cell transfection

The tRF5-GlyGCC mimic, tRF5-GlyGCC inhibitor, and their control counterparts were produced by Ribobio Co. (Guangzhou, China). The mouse sh-Runx2 plasmid was designed and produced by MIAOLING BIOLOGY (wuhan, China). Hepa1-6 cells were kept in 6-well plates to confluence for one day. The sh-Runx2 plasmid (G40989/pLV3-U6-sh-Runx2(mouse)-shRNA-CopGFP-Puro), sh-ITGBL1 plasmid (G40989/pLV3-U6-sh-ITGBL1(mouse)-shRNA-CopGFP-Puro), oe-Runx2 plasmid (pLV3-CMV-Runx (Mouse)−3 × FLAG-CopGFP-Puro), and oe-ITGBL1 plasmid (pLV3-CMV- ITGBL1 (Mouse)−3 × FLAG-CopGFP-Puro) were introduced into Hepa1-6 cells with Lipofectamine 3000 reagent following the instructions provided.

### MDSC chemotaxis assay

The MDSC chemotaxis assay was carried out in 24-well plates with Transwell chamber. In brief, MDSCs (> 90% purity) were first isolated from murine spleens, and seeded to the upper room of Transwell chamber, in the bottom room of which was added with the supernatant of Hepa1-6 cells with different treatments. After incubated for 24 h, MDSCs moving to the bottom room of Transwell chamber were counted.

### Synthesis of PAAm hydrogel

The deionized water solutions of AAm and ammonium persulfate were mixed and then heated in water bath (70℃, 30 min) to obtain PAAm hydrogel. This hydrogel was lyophilized for further use.

### Preparation and characterization of oxidized starch and Hf/TCPP NMOFs

The production and characterization of oxidized starch and hafniumions (Hf^4+^)/tetrakis (4-carboxyphenyl) porphyrin (TCPP) nanoscale metal organic framework (Hf/TCPP NMOFs) were generated according to the protocols reported in the previously published studies[9, 29].

### The efficiency of Hf/TCPP NMOFs to load tRF5-GlyGCC inhibitor

To determine the most efficient loading of tRF5-GlyGCC inhibitor, 5 μL, 10 μL, 15 μL, 20 μL, 40 μL, and 80 μL of 1 mg/mL Hf/TCPP NMOFs were mixed with 20 μL of 25 μM tRF5-GlyGCC inhibitor solution, respectively, and dispersed under sonication for half an hour to get distinct volume ratios of tRF5-GlyGCC inhibitor @Hf/TCPP NMOFs. Then, NanodropOne was applied to ascertain the free tRF5-GlyGCC inhibitor concentration in the supernatant after centrifugation at 5000 rpm for 5 min. The loading efficiency = (primary tRF5-GlyGCC inhibitor concentration—free tRF5-GlyGCC inhibitor concentration in the supernatant)/primary tRF5-GlyGCC inhibitor concentration × 100%.

### CCK-8 assay

Hepa1-6 cells were cultivated in 96-well plates under the temperature of 37 ℃ for 24 h before introducing Hf/TCPP NMOFs (0, 2.5, 5, 10, 20, 40, 50, 60, 80 μg mL^−1^) at varying concentrations. Post 24-h incubation and X-ray irradiation (4, 10 Gy), The percentage of surviving cells was assessed by utilizing a 10% CCK-8 solution (Dojindo, Japan).

### Evaluation of cell apoptosis

Hepa1-6 cells were incubated in 12-well plates, and maintained at a constant temperature of 37 ℃. Following a period of 24 h, the culture was exposed to 60 μg mL^−1^ Hf/TCPP NMOFs for an additional 24 h. Subsequently, the cells underwent X-ray exposure (4 Gy). After another 24 h,the cells were detached using trypsin and washed thoroughly. Finally, to stain the cells, an Annexin V/PI staining kit (KeyGEN BioTECH, Cat# KGA108) was employed, and flow cytometry was utilized for assessing the extent of cell apoptosis.

### Colony formation assay

This assay was followed by the previous study [30]. Hepa1-6 cells were cultured in 6-well plates. Once adhering to the bottom of plates, the tumor cells were treated with 60 μg mL^−1^ Hf/TCPP NMOFs and left to incubate for a duration of 24 h. After exposure to 4 Gy X-ray, cells were washed and cultivated with a fresh medium for another 4 days. Once sufficiently large colonies formed, the cells underwent staining with crystal violet, followed by enumeration of the viable colonies.

### Preparation and characterization of tRF5-Gi@HOP powder and its derived hydrogel

Hf/TCPP NMOFs loaded with tRF5-GlyGCC inhibitor (tRF5-Gi), OS, and freeze-dried PAAm hydrogel were mixed and ground to obtain tRF5-Gi@HOP powder, which formed hydrogel in situ rapidly when contacting PBS. The hydrogel derived from tRF5-Gi@HOP powder was subjected to analysis of its chemical structure and morphology using Fourier transform infrared spectroscopy (Nicolet 6700, Thermo Scientific) and SEM (JSM-7500F, JEOL, Japan), respectively.

### Rheological studies

The rheological evaluations were carried out in compliance with the methodologies outlined in our earlier study [8]. The strain amplitude sweep assessment (strain[γ] = 0.1–1000%) was applied to explore the critical strain threshold. In addition, for evaluating the hydrogel's self-repairing performance, an alternative step strain sweep analysis was conducted at a steady frequency of 10 radians per second.

### In vitro adhesion assays

The bonding capacity of the hydrogel derived from tRF5-Gi@HOP powder to biological substrates was assessed through the construction of lap-shear assemblies. Summarily, the hydrogel was evenly spread across two damp pieces of porcine skin (20 mm × 10 mm). The hydrogel joined the two sections of skin over an area measuring 1 cm by 1 cm. We used dynamic mechanical analysis instrument (DMA Q800, USA) executed the lap-shear examination at a progression rate of 1 mm per minute. The binding energy was quantified by executing a 180-degree peel test.

### Evaluation of biocompatibility

The assessment of hemolytic activity for tRF5-Gi@HOP powder following methodologies from existing literature. Red blood cells were obtained from whole blood via centrifugation (200 × g, 10 min), rinsed with isotonic saline solution, and then diluted to a 5% (volume/volume) concentration using the same saline solution. 50 mg of tRF5-Gi@HOP powder was introduced to the erythrocyte solutions (500 μL), and then incubated at 37 °C for a duration of 24 h. Meanwhile, we set 100 μL of isotonic saline and 0.1% Triton X-100 as negative and positive control groups, respectively. Subsequent to centrifuging the erythrocyte suspensions (500 × g) for 15 min, the supernatants were allocated into a fresh 96-well transparent plate. A microplate spectrophotometer (Thermo Scientific) was employed to gauge the absorbance at 540 nm. Hemolysis percentage (%) = (Ax − An)/(At − An) × 100% (where Ax represents the absorbance of samples with tRF5-Gi@HOP powder, An is the absorbance with isotonic saline, and At denotes the absorbance with Triton X-100). THLE-2, a normal liver cell line, was introduced into each well at a density of 25,000 cells/well, followed by an incubation period of half a day at a constant temperature of 37 °C in a 5% CO2 humidified atmosphere. Following this, 100 mg of tRF5-Gi@HOP powder was introduced into the wells harboring the cells, while a growth medium without powder served as the reference control. The percentage of viable cells was examined using the CCK8 assay and Live/Dead cell staining technique following incubation periods of 24, 48, and 72 h. Moreover, to assess biocompatibility in a living organism, tRF5-Gi@HOP powder-based hydrogel was implanted subcutaneously into mice, using PBS injections as the benchmark. Blood specimens were procured on the 3rd, 10th, and 21st days post-implantation for the biochemical examination of liver and kidney functions, alongside standard blood tests. Additionally, multiple organs of rats were collected, preserved in 4% formaldehyde, and prepared for histological analysis via H&E staining.

### The synergistic effect of RT and tRF5-Gi@HOP powder treatment in animal models

The orthotopic Hepa1-6 HCC models in Balb/c mice were first developed. On day 0, we anesthetized the mice with HCC by intraperitoneally administrating pentobarbital sodium on the same day. Then, mice were disinfected with iodophor and placed in a supine position, incising through the dermal, peritoneal, and muscular layers to disclose the left lobe of the liver. Subsequently, we inoculated 50 μL Hepa1-6 cells (1 × 10^6^ cells) into the left hepatic lobe. The establishment of orthotopic Hepa1-6 HCC within mice was verified through the use of an in vivo bioluminescence imaging technique on day 10. To explore whether tRF5-Gi@HOP powder boosts RT-elicited NK cell immunity to combat HCC recurrence postresection, those mice were randomly allocated into 5 groups after receiving hepatectomy to remove tumor masses: group 1 received no treatments (Control), group 2 for single RT (RT), group 3 for RT plus intravenous tRF5-Gi injection (RT-free-tRF5-Gi), group 4 for RT plus surgical margin-localized application of HOP powder without tRF5-Gi (RT-HOP), and group 5 for RT plus tRF5-Gi@HOP powder (RT- tRF5-Gi@HOP). The cancer recurrence was dynamically observed using the fluorescence imaging system. Observation of tumor recurrence and expansion was conducted with an in vivo bioluminescence imaging technique, as outlined earlier. The lifespan of the mice was tracked over a period of 2 months. These studies received the endorsement of the Animal Care and Use Committee of Jennio Biotech Co.,Ltd (IACUC-2024-A037).

### Flow cytometry analysis of PMN-MDSCs and NK cells in tumors

Tumor specimens were processed in complete 1640 culture medium with the addition of 0.5 mg/ml of collagenase-IV, 0.1 mg/ml of DNase-I and 0.1 mg/ml of Hyaluronidase Type V, and incubated at 37 °C for an hour. By compressing the enzymatically treated tissues through a 40 μm mesh using a syringe's plunger, a homogeneous suspension of individual cells was achieved. Then, tumor-infiltrated immune cells (TIICs) were isolated via differential speed centrifugation from single-cell suspensions. The suspensions of TIICs were subjected to flow cytometry analysis. The antibodies and dye used for flow cytometric analysis were purchased from BioLegend, including: anti-CD11b-PE (BioLegend, Cat#101,208), anti-Ly6G-BV421 (eBioscience, Cat#404–9668-80), anti-Ly6C-APC (eBioscience, Cat#17–5932-80), anti-NKp46-APC (BioLegend, Cat#137,608), anti-CD45-PE/CY7 (BioLegend, Cat#103,114), anti-GZMB-EF450 (Ebioscience, Cat#48–8898-82).

### The anti-adhesion evaluation in a hepatectomy-induced adhesion rat model

To establish the hepatectomy-induced adhesion rat model, we first performed liver resection on rats through ligating the pedicle of the median lobe using 3–0 silk sutures, then cutting the parenchyma of median lobe using surgical scissors near the base of the lobe. Similarly, the left lateral lobe was partially resected. After lavaging the peritoneal cavity with 15 mL of sterile saline, the cut surfaces of the excisional liver parenchyma were completely covered with tRF5-Gi@HOP powder or Biopolysaccharide flushing gum solution, a common commercial anti-adhesion biomaterial. Concerning control group, we only closed the peritoneal cavity without any anti-adhesion biomaterials applied. After 7 or 14 days of treatment, we euthanized the rats and opened their abdomen to assess and score the tissue adhesion status as previously described [5].

### Statistical analysis

All the data were analyzed at the help of GraphPad Prism V.7 and IBM SPSS Statistics V.20. The statistical test methodologies applied for analyzing data covered the log-rank test, Student’s t test, and One-way repeated-measures ANOVA test. The Kaplan–Meier analysis was implemented to evaluate the survival outcome of mice. p < 0.05 indicated significance (see Fig. 1).

## Results and discussion

### RT-induced glycocholic acid impairs NK cell cytotoxicity in HCC

**Fig. 2:**
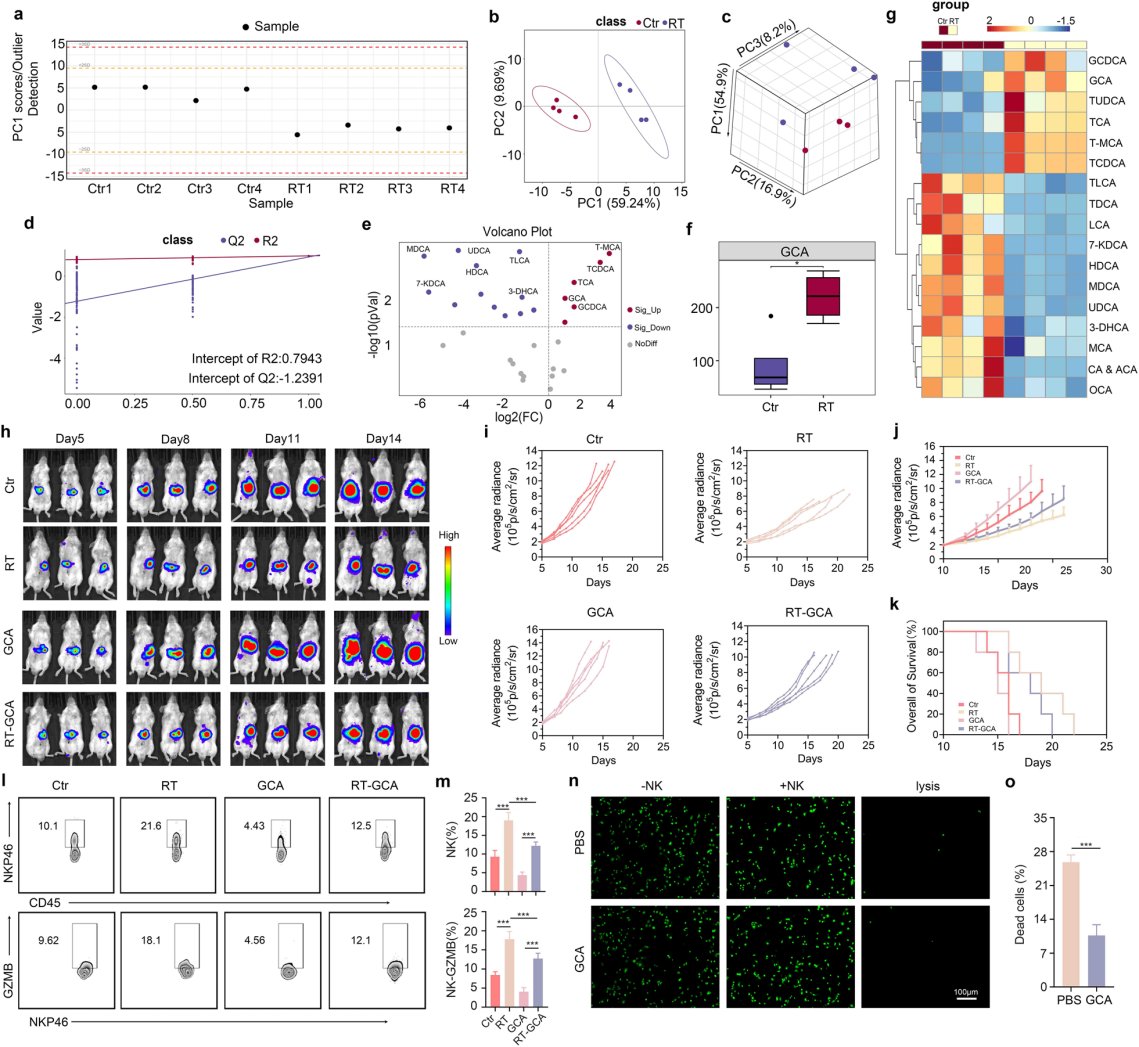
RT-induced glycocholic acid impairs NK cell cytotoxicity in HCC. **a** The PC1 scores in Multivariate Control Chart (MCC) for BA samples of mouse HCC tissues in control group and RT group. **b**, **c** The OPLS-DA model was adopted for evaluating the classification of BA samples of mouse HCC tissues in control group and RT group. **d:** The permutation test of OPLS-DA model. **e–g** The BA profiles in control group and RT group are significantly different. **h**-**j** Glycocholic acid (GCA) substantially limits RT efficacy in mouse HCC model in terms of tumor growth control. n = 5 **k**: GCA markedly limits RT efficacy in terms of survival time of mice. **l**–**m** GCA dramatically attenuates the effect of RT in enhancing NK cell infiltration and activity in mouse HCC. **n**–**o** GCA significantly promotes Hepa1-6 cells to resist NK cell-meditated cell lysis. n = 3. Data shown as means ± SD. P values of k wrer performed by the Kaplan–Meier analysis. P values of m were calculated using a One-way repeated measures ANOVA test. P values of o were calculated using a two-sided unpaired Student’s t test

To explore whether BAs are involved in the RT-mediated inhibition of NK cells, UPLC-MS was used to measure the concentrations of 50 BAs in mouse HCC tissues of the control and RT groups. As Fig. 2a shows, none of the PC1 scores in the multivariate control chart exceeded 10 standard deviations, indicating that the sample preparation was performed under good quality control. The OPLS-DA model was used to evaluate the classification of tissue samples in terms of BA profiles. The results showed that samples from the control group could be significantly distinguished from those from the RT group (Fig. 2b, c). The permutation test indicated that the classification model was valid (Fig. 2d). As Fig. 2e–g show, RT substantially altered BA profiles in mouse HCC tissues. Six types of BAs were highly expressed in the RT group compared to the control group. Among the upregulated BAs, glycocholic acid (GCA) was associated with HCC progression. Therefore, we explored whether GCA was involved in the RT-mediated suppression of NK cell antitumor immunity in HCC. We treated orthotopic Hepa1-6 tumor-bearing mice with PBS, RT, intraperitoneal GCA injection, or RT combined with intraperitoneal GCA injection. Tumor growth was monitored using in vivo bioluminescence imaging. As Fig. 2h–k show, GCA treatment dramatically abrogated RT efficacy in the mouse HCC models. Moreover, RT significantly increased the frequency and activity of NK cells in HCC, and this effect was markedly reversed by GCA treatment (Fig. 2l–m). Further experiments showed that pre-treatment with GCA drastically boosted the resistance of HCC cells to NK cell-mediated cell lysis (Fig. 2n, o). Altogether, these results suggest that GCA participates in the RT-mediated inhibition of NK cell cytotoxicity in HCC.

### GCA inhibits NK cell cytotoxicity by activating the tRF5-GlyGCC/Runx2/ITGBL1 pathway in HCC cells

**Fig. 3:**
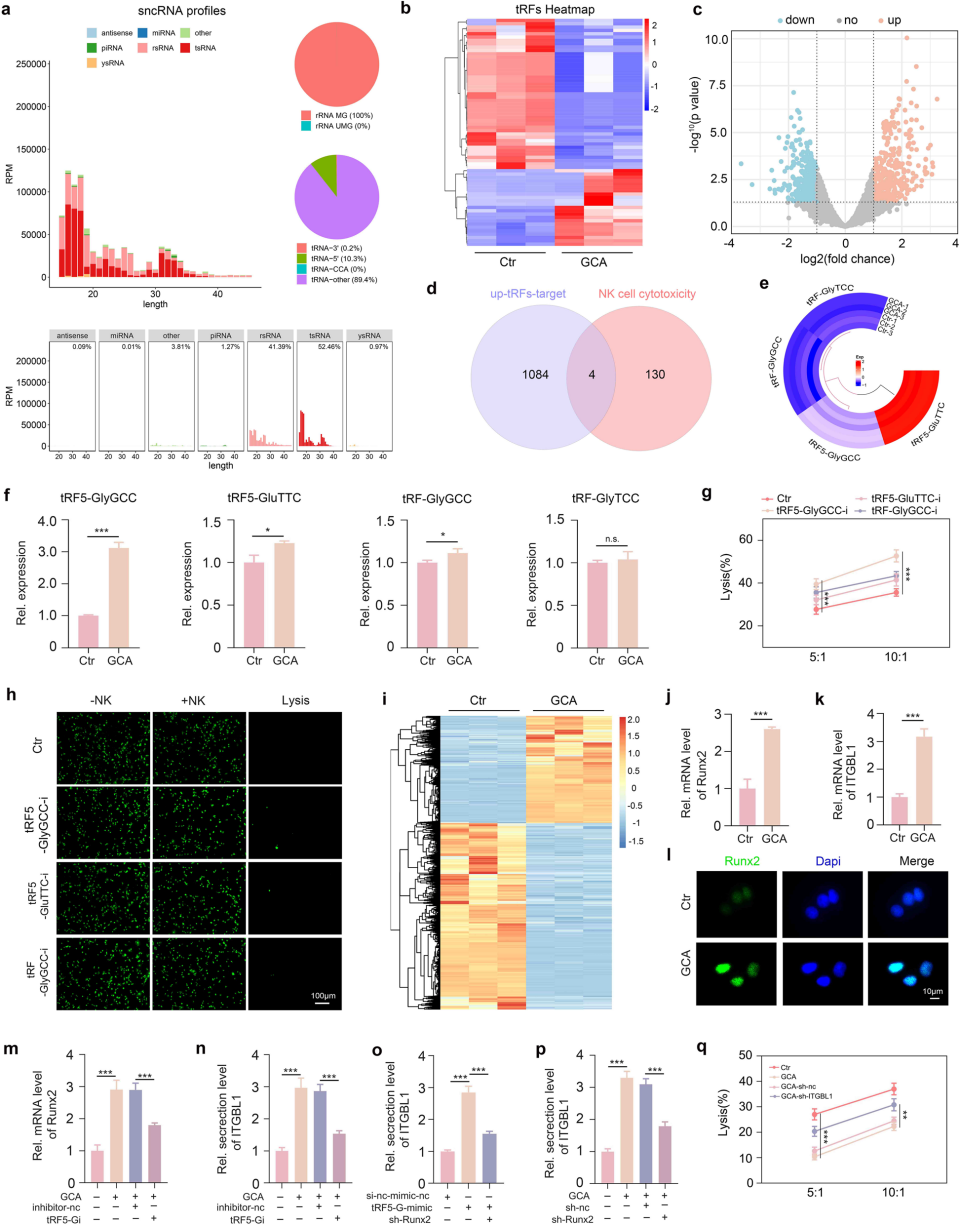
GCA inhibits NK cell cytotoxicity by activating tRF5-GlyGCC/Runx2/ITGBL1 pathway in HCC cells. **a** The abundance of sncRNAs differently expressed in GCA-treated cells. **b**, **c** The differently expressed tRFs between GCA-treated cells and control cells. **d** The intersection of the target genes of top 50 upregulated tRFs in GCA-treated cells with NK cell cytotoxicity-related genes. **e** The intersected genes in (**d**) are targeted by 4 upregulated tRFs. **f** qPCR validation of the 4 tRFs. **g** Lactate dehydrogenase release assay for evaluating the effects of different tRFs on Hepa1-6 cell sensitivity to NK cell killing. **h** Calcein AM staining assay for evaluating the effects of different tRFs on Hepa1-6 cell sensitivity to NK cell killing. **i** The differently expressed mRNA profiles between GCA-treated and control groups. **j** qPCR analysis of GCA-meditated effect on Runx2 expression. **k** qPCR analysis of GCA-meditated effect on ITGBL1 expression. **l** Immunochemistry staining analysis of Runx2 expression. **m** qPCR analysis of the effect of tRF5-G1yGCC inhibitor on GCA-stimulated Runx2 expression. **n** ELISA analysis of the effect of tRF5-G1yGCC inhibitor on GCA-stimulated ITGBL1 expression.** o** ELISA analysis of ITGBL1 secretion in different groups. **p** ELISA analysis of the effect of Runx2 knockdown on GCA-stimulated ITGBL1 secretion. **q** Lactate dehydrogenase release assay evaluating the effect of ITGBL1 knockdown on GCA-meditated Hepa1-6 cell resistance to NK cell killing. Data shown as means ± SD. n = 3. The P values of f, j and k were calculated using a two-sided unpaired Student’s t test. P values of g, m, n, o, p and q were calculated using a One-way repeated measures ANOVA test

tRNA-derived fragments (tRFs) are a newly identified class of small noncoding RNAs (sncRNAs) produced via the specific cleavage of mature or precursor transfer RNAs [23]. Over the past few decades, tRFs have emerged as essential players in the complex network of cancer biology and are involved in cancer growth, metastasis, immune escape, and therapy resistance [31]. In the current study, we investigated whether GCA is involved in the resistance of HCC cells to NK cell killing. Accordingly, we first used PANDORA-seq technology to compare small non-coding RNA (sncRNA) profiles in GCA-treated and control HCC cells. As Figs. 3a–c and S1 show, tRFs in Hepa1-6 cells were the most abundant among all sncRNAs, and a large number of tRFs were differentially expressed in GCA-treated and control cells. We then intersected the target genes of the top 50 upregulated tRFs with those associated with NK cell cytotoxicity, identifying four target genes (Fig. 3d) that were targeted by the four upregulated tRFs (Fig. 3e). qPCR validation of these tRFs showed that only tRF5-G1yTCC, tRF5-GluTCC, and tRF-GlyGCC were significantly upregulated in GCA-treated HCC cells (Fig. 3f). Moreover, lactate dehydrogenase (LDH) release and calcein AM staining experiments indicated that the tRF5-GlyGCC inhibitor (tRF5-Gi) had the strongest capacity to sensitize Hepa1-6 cells to NK cell-mediated lysis (Figs. 3g–h and S2). Accordingly, we further investigated how tRF5-GlyGCC regulated Hepa1-6 cell sensitivity to NK cell death. For this purpose, we first applied high-throughput sequencing to compare the mRNA profiles of GCA-treated Hepa1-6 and control cells. As Figs. 3i and S3 show, GCA significantly altered the mRNA profiles of Hepa1-6 cells. ITGBL1 has been revealed to be an inhibitory factor of NK cell cytotoxicity, and Runx2 is a crucial transcription factor that directly activates ITGBL1 expression in a variety of cells, including tumor cells [25]. Coincidently, our mRNA sequencing results showed that Runx2 and ITGBL1 were simultaneously upregulated in GCA-treated Hepa1-6 cells. These findings suggest that tRF5-GlyGCC in Hepa1-6 cells may disturb NK cell cytotoxicity via the Runx2/ITGBL1 signaling axis, thereby reducing the sensitivity of Hepa1-6 cells to NK cell killing. To test this hypothesis, we first evaluated the expression levels of Runx2 and ITGBL1 in Hepa1-6 cells. As Fig. 3j–k show, Runx2 and ITGBL1 mRNA levels were significantly elevated after GCA treatment. GCA consistently increased the protein expression of Runx2 and ITGBL1 (Figs. 3l and S4). In addition, the tRF5-GlyGCC inhibitor (tRF5-Gi) markedly reversed the GCA-induced expression of Runx2 and ITGBL1 (Fig. 3m, n). Similar to GCA treatment, tRF5-GlyGCC mimics substantially promoted Hepa1-6 cells to express ITGBL1, whereas knockdown of Runx2 significantly abrogated this effect (Fig. 3o, p). Importantly, ITGBL1 knockdown substantially attenuated the resistance of GCA-induced HCC cells to NK cell-induced lysis (Fig. 3q). Collectively, GCA inhibited NK cell cytotoxicity via the tRF5-GlyGCC/Runx2/ITGBL1 signaling axis in HCC cells.

### GCA promotes MDSC recruitment to orchestrate tumor immunosuppression via the tRF5-GlyGCC/Runx2/S100A9 axis in HCC cells

**Fig. 4:**
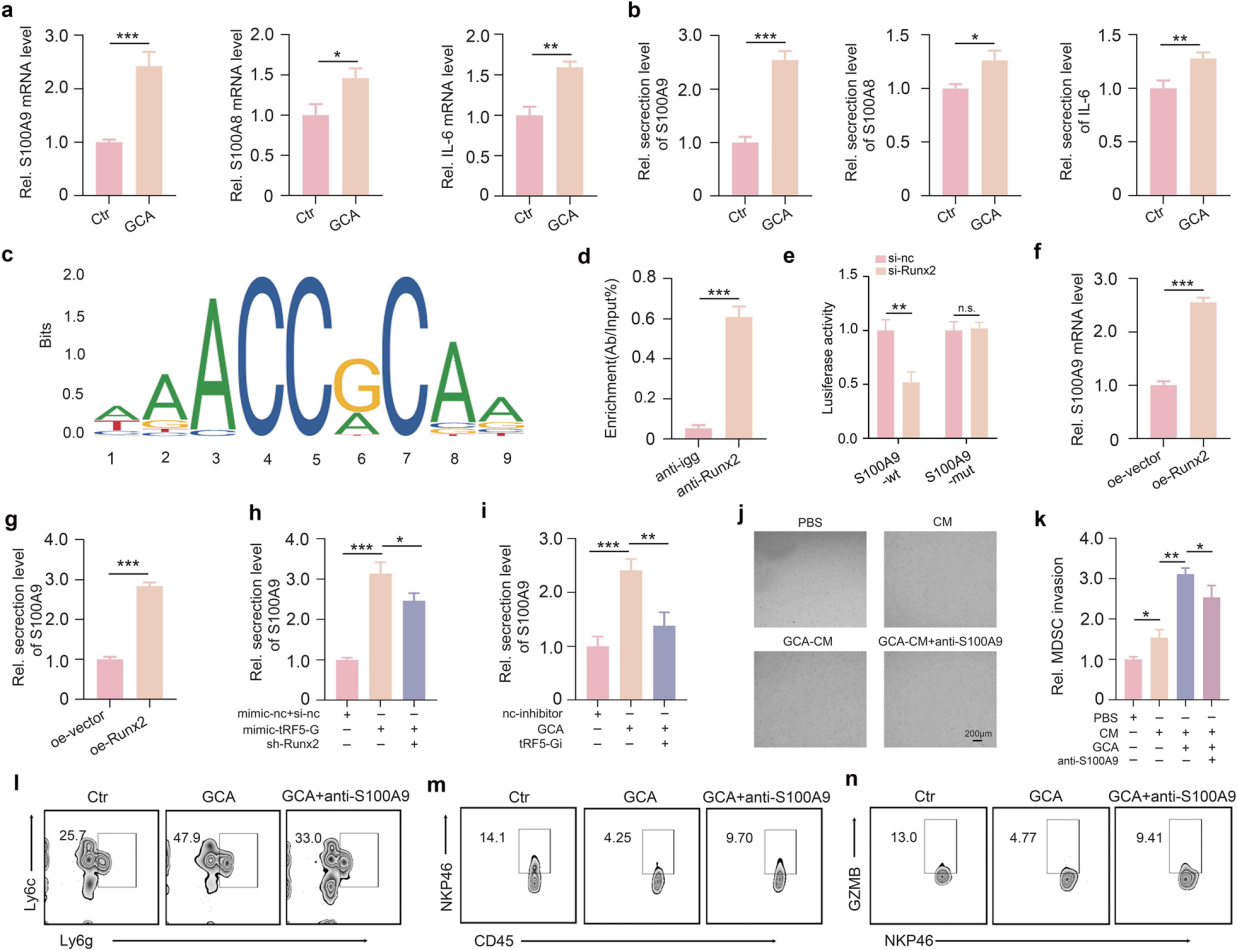
GCA promotes MDSC recruitment to orchestrate immunosuppression via tRF5-GlyGCC/Runx2/S100A9 axis in HCC cells. **a** qPCR analysis of GCA-meditated effect on S100A9, S100A8, and IL-6 expressions in Heapa1-6 cells. **b** ELISA analysis of S100A9, S100A8, and IL-6 secretion from GCA-treated Heapa1-6 cells. **c** The putative Runx2-binding sites in the S100A9 promoter. **d** ChIP-PCR assay showed that Runx2 directly bound to the S100A9 promoter. **e** Luciferase reporter assay showed the binding of Runx2 to the S100A9 promoter. **f** qPCR analysis of the effect of Runx2 in regulating S100A9 expression. **g** ELISA analysis of the effect of Runx2 in regulating S100A9 expression. **h** ELISA analysis of S100A9 secretion in distinct groups. **i** ELISA analysis of the effect of tRF5-G1yGCC inhibitor on GCA-stimulated S100A9 expression. **j**–**k** The transwell assay assessing the effect of anti-S100A9 antibody on the recruitment of murine PMN-MDSCs. **l** Anti-S100A9 antibody treatment increased the frequency of tumor-infiltrating PMN-MDSCs in vivo. **m**, **n** The number and activity of NK cells in mouse HCC tissues of distinct groups. Data shown as means ± SD. n = 3. P values of a, b, d, e, f and g were calculated using a two-sided unpaired Student’s t test. P values of h, i and k were calculated using a One-way repeated measures ANOVA test

In addition to the direct inhibition of NK cell cytotoxicity, tumor cells have also been reported to indirectly impair NK cell cytotoxicity through polymorphonuclear myeloid-derived suppressor cells (PMN-MDSCs) [32]. Our mRNA-seq analysis showed that GCA increased the expression levels of several PMN-MDSC chemokines, including IL-6, S100A8, and S100A9, in Hepa1-6 cells (Fig. S3). Furthermore, PCR and ELISA confirmed that S100A9 was significantly upregulated after GCA treatment (Fig. 4a, b). Interestingly, prediction based on JASPAR database analysis identified several putative Runx2-binding sites in the S100A9 promoter (Figs. 4c and S5). Therefore, we investigated whether GCA facilitates PMN-MDSC recruitment to the tumor microenvironment via the tRF5-GlyGCC/Runx2/S100A9 signaling axis in HCC cells. To answer this issue, the Runx2-mediated transcriptional regulation of S100A9 expression was explored. A ChIP-PCR assay showed that Runx2 directly bound to the S100A9 promoter (Fig. 4d). Next, we cloned a 2.0-kb region of the S100A9 promoter into the pGL4.10 vector to generate the luciferase reporter gene plasmid, which was then co-transfected into Hepa1-6 cells with the Runx2 knockdown plasmid or control plasmid. Figure 4e shows that there was much weaker luciferase activity in cells co-transfected with the plasmid containing the wild-type S100A9 promoter and Runx2 knockdown plasmid than in cells co-transfected with the plasmid containing the mutated S100A9 promoter and control plasmid. Moreover, PCR and ELISA analyses showed that Runx2 overexpression significantly upregulated S100A9 expression in Hepa1-6 cells (Fig. 4f–g). These data indicated that S100A9 is a direct target of Runx2 in HCC cells. In addition, we found that tRF5-GlyGCC mimics prominently promoted Hepa1-6 cells to secrete S100A9, but this effect was attenuated by Runx2 knockdown (Fig. 4h). Moreover, tRF5-Gi markedly mitigated the GCA-stimulated upregulation of S100A9 in Hepa1-6 cells (Fig. 4i). These results suggest that GCA can stimulate S100A9 secretion via activation of the tRF5-GlyGCC/Runx2 pathway. As expected, the GCA-treated Hepa1-6 cell-conditioned medium was found to significantly attract murine PMN-MDSCs in vitro, whereas the anti-S100A9 antibody drastically blocked this process (Fig. 4j–k). Consistent with these results, GCA treatment substantially increased the frequency of tumor-infiltrating PMN-MDSCs in orthotopic Hepa1**-**6 mouse models, which was markedly reversed by anti-S100A9 antibody treatment (Figs. 4l and S6a). Furthermore, we found that the number of total and activated NK cells in mouse HCC tissues was markedly decreased after GCA treatment, and anti-S100A9 antibody treatment significantly mitigated this effect (Fig. 4m, n and S6b–c). Taken together, these data suggest that GCA promotes PMN-MDSC recruitment to orchestrate tumor immunosuppression via the tRF5-GlyGCC/Runx2/S100A9 axis in HCC cells, thereby impairing NK cell cytotoxicity.

### tRF5-GlyGCC interacts with KDM6B to epigenetically activate Runx2 transcription

**Fig. 5:**
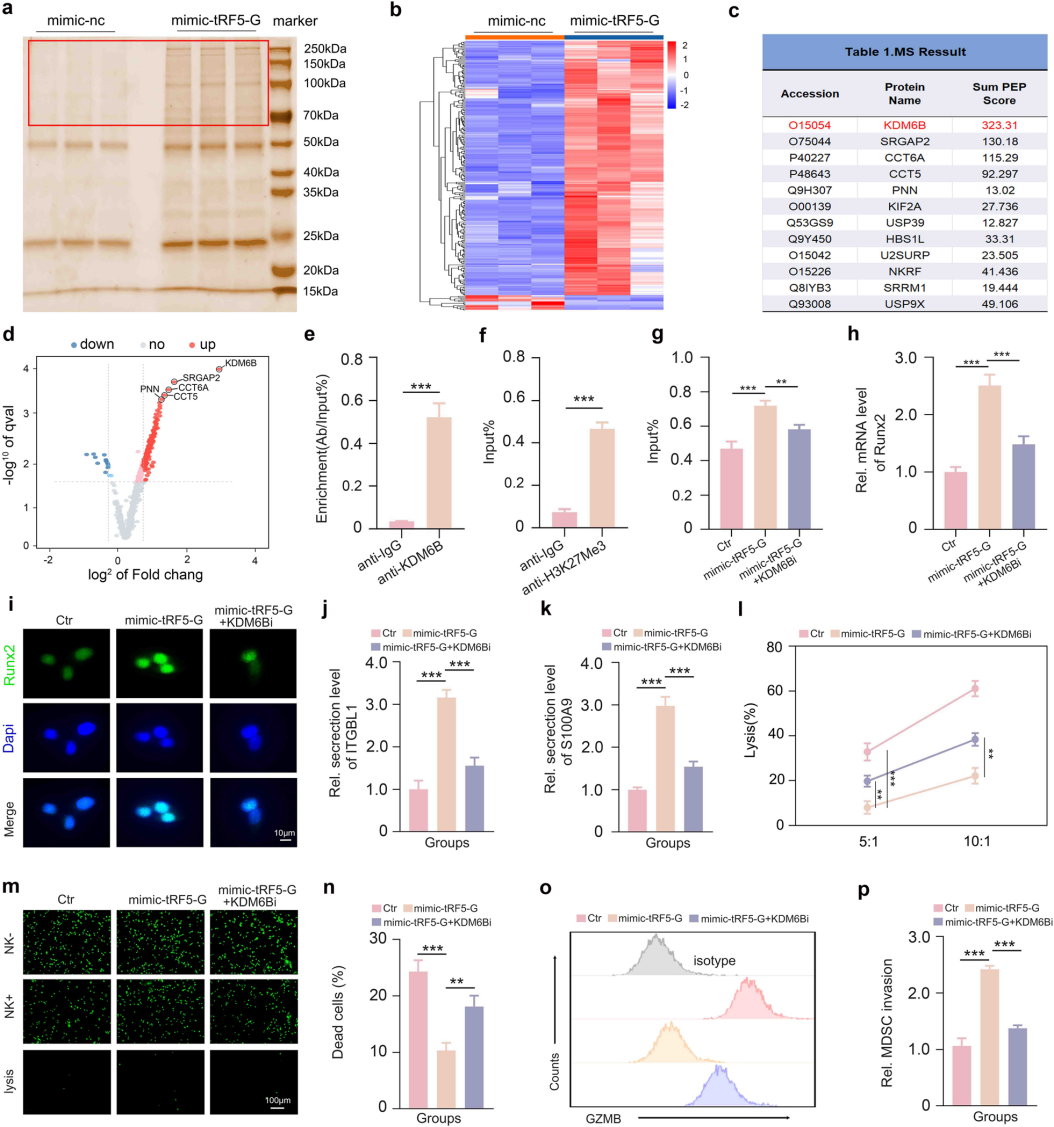
tRF5-GlyGCC interacts with KDM6B to epigenetically activate Runx2 transcription. **a** Silver SDS-PAGE gel image uncovered proteins immunoprecipitated by tRF5-GlyGCC and its antisense RNA in Hepa1-6 cells. **b-d** tRF5-GlyGCC binding proteins with top matching scores identified by mass spectrometry. **e** RIP assay confirmed the binding of tRF5-GlyGCC to KDM6B. **f** ChIP-PCR assay showed that the trimethylation of histone H3 lysine 27 existed on the Runx2 promoter. **g** ChIP-PCR assay assessing the trimethylation of histone H3 lysine 27 on the Runx2 promoter in different groups. **h** qPCR analysis of the effect of KDM6B mimics on tRF5-GlyGCC mimic-stimulated Runx2 expression. **i** Immunofluorescence staining analysis of Runx2 expression in distinct groups. **j** ELISA analysis of ITGBL1 expression in different groups. **k** ELISA analysis of S100A9 expression in different groups. **l**: Lactate dehydrogenase release assay for evaluating the effect of HCC cell resistance to NK cell killing. **m**, **n** Calcein AM staining assay for evaluating the effect of HCC cell resistance to NK cell killing. **o** The GZMB levels of different groups.** p** The ability of HCC cells in different groups to attract PMN-MDSCs. Data shown as means ± SD. n = 3. P values of e and f were calculated using a two-sided unpaired Student’s t test. P values of g, h, j, k, l, n and p were calculated using a One-way repeated measures ANOVA test

Evidence shows that tRFs perform biological functions by interacting with proteins [31]. Therefore, we conducted RNA pull-down experiments followed by mass spectrometry to elucidate the mechanisms by which tRF5-GlyGCC upregulates Runx2 expression in Hepa1**-**6 cells (Fig. 5a). We observed that tRF5-GlyGCC likely interacts with multiple cancer-associated RNA-binding proteins, including KDM6B, SRGAP2, CCT6A, CCT5, and USP9X (Fig. 5b–d). Among these proteins, we showed much interest in KDM6B, since it obtained the highest matching score and has been shown to be closely correlated with the aggravation of HCC [33, 34]. Using the RIP assay, we further validated the binding of tRF5-GlyGCC to KDM6B in Hepa1**-**6 cells (Fig. 5e). It is well established that KDM6B mediates histone 3 lysine 27 (H3K27) demethylation, which is an important epigenetic mechanism that drives gene activation [35]. Thus, we hypothesized that tRF5-GlyGCC may regulate Runx2 expression in a KDM6B-dependent manner. To test this hypothesis, we conducted a ChIP-PCR assay to examine the trimethylation of histone H3 lysine 27 (H3K27me3) in the Runx2 promoter. Figure 5f–g show that tRF5-GlyGCC inhibitor significantly decreased the H3K27me3 level of the Runx2 promoter, whereas the KDM6B inhibitor markedly reversed this effect. Next, we examined the regulatory effect of the tRF5-GlyGCC/KDM6B signaling axis on Runx2 expression in Hepa1**-**6 cells. As Fig. 5h–i show, the KDM6B inhibitor substantially counteracted the tRF5-GlyGCC mimic-induced upregulation of Runx2. A previous study has shown that some tRFs can directly interact with epigenetic enzymes and then regulate their expression or recruitment to the promoters of their targets, thereby exerting diverse biological functions [31]. Notably, our WB assay showed that tRF5-GlyGCC mimics had no significant effect on KDM6B expression in Hepa1**-**6 cells (Fig. S7), indicating that tRF5-GlyGCC upregulated Runx2 expression by recruiting KDM6B to the Runx2 promoter. Next, we showed that the KDM6B inhibitor significantly attenuated the GCA-induced activation of the Runx2/ITGBL1/S100A9 signaling axis in Hepa1**-**6 cells (Fig. 5j–k). As expected, the LDH release and calcein AM staining assays showed that the KDM6B inhibitor dramatically mitigated the effect of tRF5-GlyGCC mimics in reducing Hepa1**-**6 cell cytotoxicity in NK cells (Fig. 5l–n). In addition, NK cells co-cultured with Hepa1**-**6 cells treated with the tRF5-GlyGCC mimic plus KDM6B inhibitor showed much lower cytotoxicity than cells co-cultured with tRF5-GlyGCC mimic-transfected Hepa1**-**6 cells (Fig. 5o). Moreover, HCC cells treated with tRF5-GlyGCC mimics and a KDM6B inhibitor had a significantly lower ability to attract PMN-MDSCs in vitro than cells transfected with tRF5-GlyGCC mimics (Figs. 5p and S8). Taken together, these results suggest that tRF5-GlyGCC interacts with KDM6B to epigenetically activate Runx2 expression, thereby boosting the secretion of ITGBL1 and S100A9 by HCC cells to impair NK cell antitumor immunity.

### Preparation and characterization of tRF5-Gi@HOP powder and its derived hydrogel

**Fig. 6:**
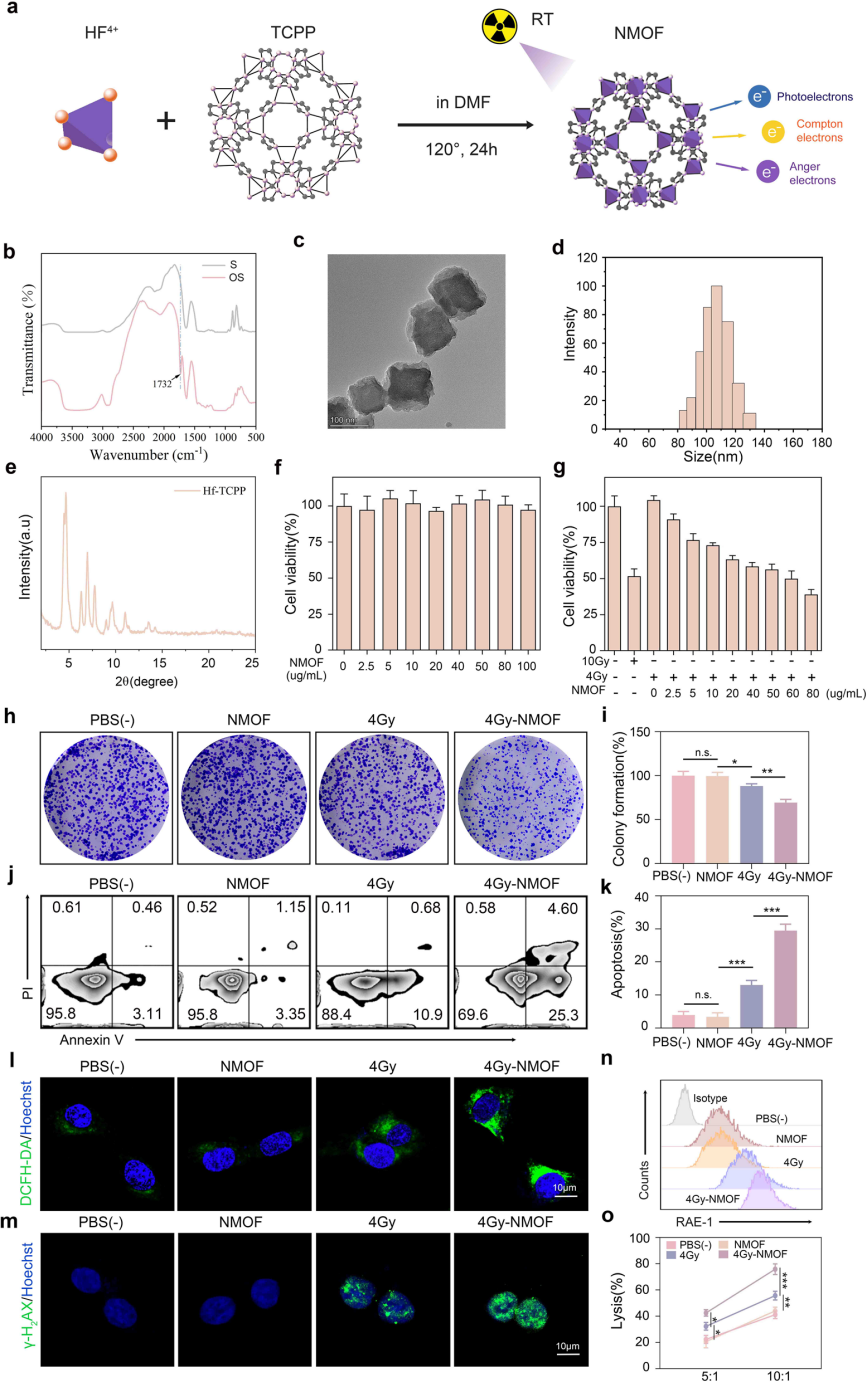
Synthesis of Hf-TCPP NMOFs and oxidized starch. **a** The schematic illustrating the synthesis of NMOFs and X-ray absorbance by Hf for enhanced RT. **b** Fourier transform infrared (FTIR) analysis of oxidized starch. **c**: TEM image of Hf-TCPP NMOFs. **d** Malvern Nano zetasizer (UK) analysis of average hydrodynamic size of Hf-TCPP NMOFs. **e** PXRD patterns of NMOFs.** f** Survival rates of Hepa1-6 cells after exposure to different concentrations of Hf-TCPP NMOFs. **g** Survival rates of HCC cells after exposure to different concentrations of Hf-TCPP NMOFs under 4 or 10 Gy X-ray irradiation. **h**–**i** Colony formation capacity of Hepa1-6 cells treated by Hf-TCPP NMOFs combined with RT or not. **j–k** Apoptosis of Hepa1-6 cells treated by Hf-TCPP NMOFs combined with RT or not. **l** Confocal microscopy visuals showcasing ROS level in different groups. **m** γ-H2AX staining for DNA fragmentation of distinct groups. **n** Flow cytometry analysis of REA-1 expression of different groups. **o** Lactate dehydrogenase release assay for assessing the sensitivity of Hepa1-6 cells in different groups to NK cell killing. Data shown as means ± SD. n = 3. P values of i, k and o were calculated using a One-way repeated measures ANOVA test

To target tRF5-GlyGCC to alleviate RT-mediated inhibition of NK cell antitumor immunity in HCC, we developed a self-gelation powder for sustained surgical margin-localized delivery of tRF5-Gi. We first produced OS by oxidizing starch with sodium periodate according to a previously described protocol [36]. Fourier transform infrared (FTIR) analysis revealed a newly emerging infrared band at 1732 cm^−1^ in the spectrum after oxidation (Fig. 6b), which was caused by the stretching vibration of the aldehyde groups. Next, we mixed and heated ammonium persulfate and the deionized water solutions of AAm in water bath (70 ℃, 30 min) to generate PAAm hydrogel. Hf/TCPP nanoscale MOFs (NMOFs) were produced as described in a previous study [29] (Fig. 6a). Transmission electron microscopy analysis proved that the Hf/TCPP NMOFs displayed a microspherical shape with a diameter of approximately 110 nm (Fig. 6c), which was further validated by Malvern Zetasizer Nano analysis (Fig. 6d). X-ray diffraction confirmed the highly crystalline structure of the nanoparticles (Fig. 6e). Interestingly, it has been revealed that the interaction between X-rays and high-Z elements can trigger the release of Compton electrons, Auger electrons, and photoelectrons, and then these electrons are arrested by H_2_O molecules in the tumor microenvironment to generate cytotoxic hydroxyl radicals, resulting in aggravated DNA damage to amplify RT-induced apoptosis [30]. Accordingly, we investigated whether the Hf/TCPP NMOFs could sensitize HCC cells to RT-mediated cytotoxicity. CCK-8 assays showed that incubation with Hf/TCPP NMOFs for 24 h without X-ray exposure resulted in slight toxicity to Hepa1-6 cells (Fig. 6f). However, X-ray exposure (4 or 10 Gy) caused substantial cytotoxicity, and the Hf/TCPP NMOFs enhanced this effect in a concentration-dependent manner (Fig. 6g). Considering that 60 μg mL^−1^ Hf/TCPP NMOFs with 4 Gy X-ray irradiation resulted in approximately 50% cell death, this treatment protocol was applied to perform subsequent experiments, such as colony formation and apoptosis assays. The results showed that Hf/TCPP NMOFs alone exerted no significant effect on colony formation or apoptosis in Hepa1-6 cells (Fig. 6h–k). However, they can dramatically sensitize RT cells to inhibit HCC cell colony formation and induce apoptosis. ROS-triggered DNA damage is a crucial mechanism underlying RT-induced cytotoxicity [37]. Therefore, we further took advantage of 2′,7′-dichlorodihydro-fluorescein diacetate, a green fluorogenic probe, to detect the ROS levels in Hepa1-6 cells. As presented in Fig. 6l, much more intense green fluorescence was detected in cells exposed to X-rays and Hf/TCPP NMOFs than in cells exposed to X-rays alone, indicating that Hf/TCPP NMOFs can augment RT to stimulate ROS production in HCC cells. Consistently, our immunofluorescence assay showed that green γ-H2AX-positive foci were substantially increased in Hepa1-6 cells co-treated with Hf/TCPP NMOFs and X-rays, which implied that Hf/TCPP NMOFs augmented RT-elicited DNA damage (Fig. 6m). Previous studies have verified that RT-induced DNA damage can stimulate the expression of RAE-1, an NKG2D ligand, in tumor cells, resulting in increased sensitivity to NK cell killing [38]. Consistent with this, our data showed that RT significantly increased RAE-1 expression in Hepa1-6 cells (Fig. 6n) and sensitized Hepa1-6 cells to NK cell-mediated cell lysis (Fig. 6o), which was further enhanced by the Hf/TCPP NMOFs. These results suggest that the Hf/TCPP NMOFs enhance the sensitivity of HCC cells to RT-induced cytotoxicity and NK cell-mediated cell lysis. To evaluate the capacity of the Hf/TCPP NMOFs to load tRF5-Gi, we investigated the effect of the volume ratio (NMOFs/tRF5-Gi) on the loading efficiency at specific concentrations (NMOFs: 1 mg/mL, tRF5-Gi: 5 nM). As presented in Fig. S9, tRF5-Gi was completely encapsulated in the NMOFs when the volume ratio reached 2 or higher. These results imply that Hf/TCPP NMOFs have an excellent ability to load tRF5-Gi.

**Fig. 7:**
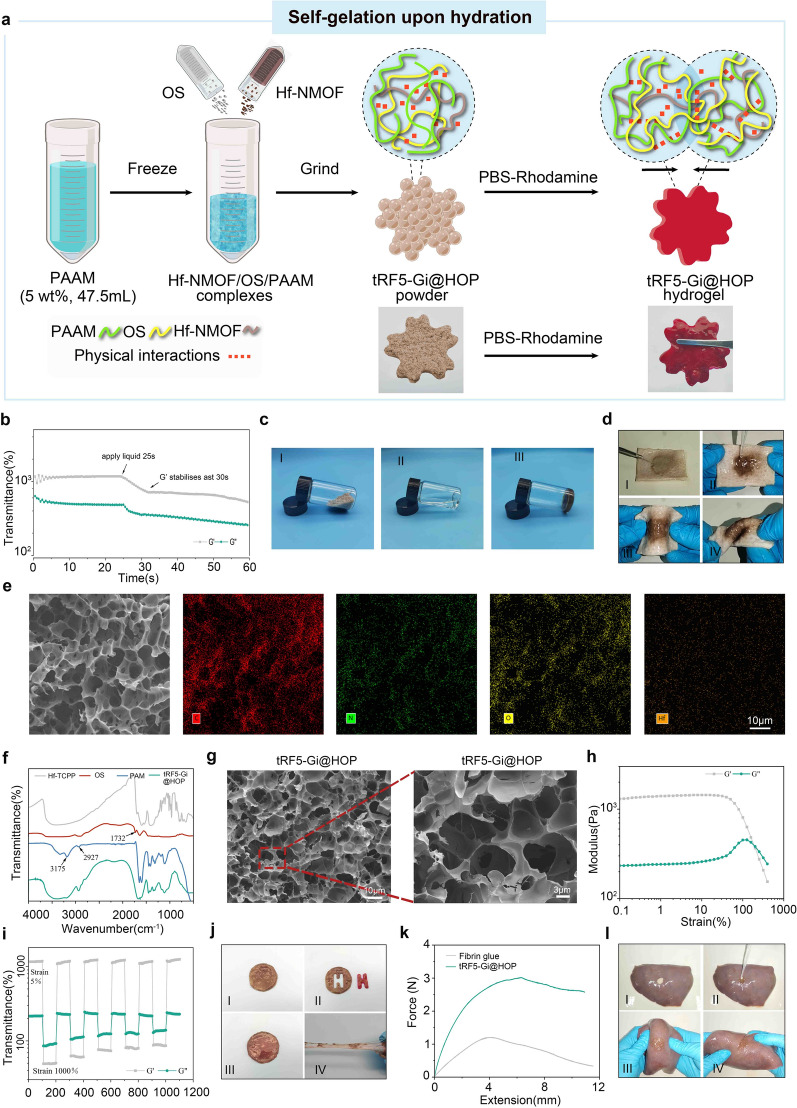
Synthesis and characterization of tRF5-Gi@HOP powder and its derived hydrogel. **a** The diagram summarizing the process of fabricating tRF5-Gi@HOP powder and its derived hydrogel. **b** The gelation time of tRF5-Gi@HOP powder. **c** tRF5-Gi@HOP powder forms hydrogel in a glass bottle after adding water. **d** The tRF5-Gi@HOP powder sprayed on fresh pork skin rapidly forms adhesive hydrogel in situ, once water is added. **e** Element mapping analysis of the composition of tRF5-Gi@HOP powder-derived hydrogel. **f** Fourier transform infrared analysis of the composition of tRF5-Gi@HOP powder-derived hydrogel. **g** The scanning electron microscopy analysis of tRF5-Gi@HOP powder-derived hydrogel. **h** The strain amplitude sweep evaluation of tRF5-Gi@HOP powder at a consistent angular velocity. **i** The continuous cyclic strain test of tRF5-Gi@HOP powder-derived hydrogel. **j** The hydrogel chiselled into two parts could fast self-integrate into a single entity. **k** Lap shear tests of tRF5-Gi@HOP powder-derived hydrogel and fibrin glue. **l** Macroscopic test of the adhesion of tRF5-Gi@HOP powder-derived hydrogel to fresh porcine liver

Subsequently, the Hf/TCPP NMOFs encapsulating tRF5-Gi, OS, and PAAm hydrogels were fully mixed and ground to generate a nanocomposite powder, hereafter referred to as the tRF5-Gi@HOP powder (Fig. 7a). Upon contact with water, the tRF5-Gi@HOP powder rapidly formed a stable hydrogel in situ (Fig. 7b–d and Movie S1). The amino groups in PAAm can react with the aldehyde and carboxyl groups in OS to yield dynamic covalent Schiff’s base bonds and reversible hydrogen bonds, respectively, which may chiefly account for the self-gelation of the tRF5-Gi@HOP powder [9]. Element mapping analysis showed that C, N, O, and Hf were present in the tRF5-Gi@HOP powder-derived hydrogel, indicating the composition of PAAm, OS, and Hf/TCPP NMOFs (Fig. 7e), which was further confirmed by the FTIR analytical results (Fig. 7f). Scanning electron microscopy revealed the porous architecture of the tRF5-Gi@HOP powder-derived hydrogel (Fig. 7g). The strain amplitude sweep test showed that the hydrogel's G′ and G′′ moduli were steady up to a 10% strain level (Fig. 7h), suggesting that the hydrogel had a potent capacity for elastic deformation. When the strain was elevated to approximately 200%, the hydrogel's G′ and G′′ became equal, indicating that the hydrogel was transformed into a solution state. The self-healing potency of the tRF5-Gi@HOP powder-derived hydrogel was determined by a cyclic strain experiment in which the strains were cycled between 5 and 1000% at a stable angular frequency of 1 rad/s. Figure 7i shows that the hydrogel fell sharply into the solution state as soon as the strain reached 1000%. When the strains declined back to 5%, the G′ and G′′ moduli were abruptly recovered to their initial values without any loss as the solution reverted into the hydrogel. We then performed a macroscopic self-amending test to further investigate the self-healing ability of the hydrogel. As Fig. 7j illustrates, the hydrogel was chiseled into two parts and quickly self-integrated into a single entity within 5 min without any additional stimulus. These results support that the tRF5-Gi@HOP powder-derived hydrogel has good self-healing performance. Wet-tissue adhesion is crucial for the postsurgical liver-localized drug delivery platform; therefore, we conducted a lap shear test to estimate the wet-tissue adhesion performance of the tRF5-Gi@HOP powder-derived hydrogel. As Fig. 7k shows, the hydrogel had much higher adhesive strength than the fibrin glue. Moreover, Fig. 7l and Movie S2 show that the hydrogel tightly adhered to fresh liver tissues, regardless of repeated twisting, bending, or flushing with PBS. Altogether, these data demonstrate that the tRF5-Gi@HOP powder-derived hydrogel displays strong wet-tissue adhesion capacity. Mechanistically, the dynamic covalent Schiff base bond formed by the reaction of the aldehyde groups in the OS and the amino groups in the wet tissue substrates may primarily confer wet tissue adhesion to the hydrogel [8].

### Biocompatibility, biodegradation and drug release behavior of tRF5-Gi@HOP powder

**Fig. 8:**
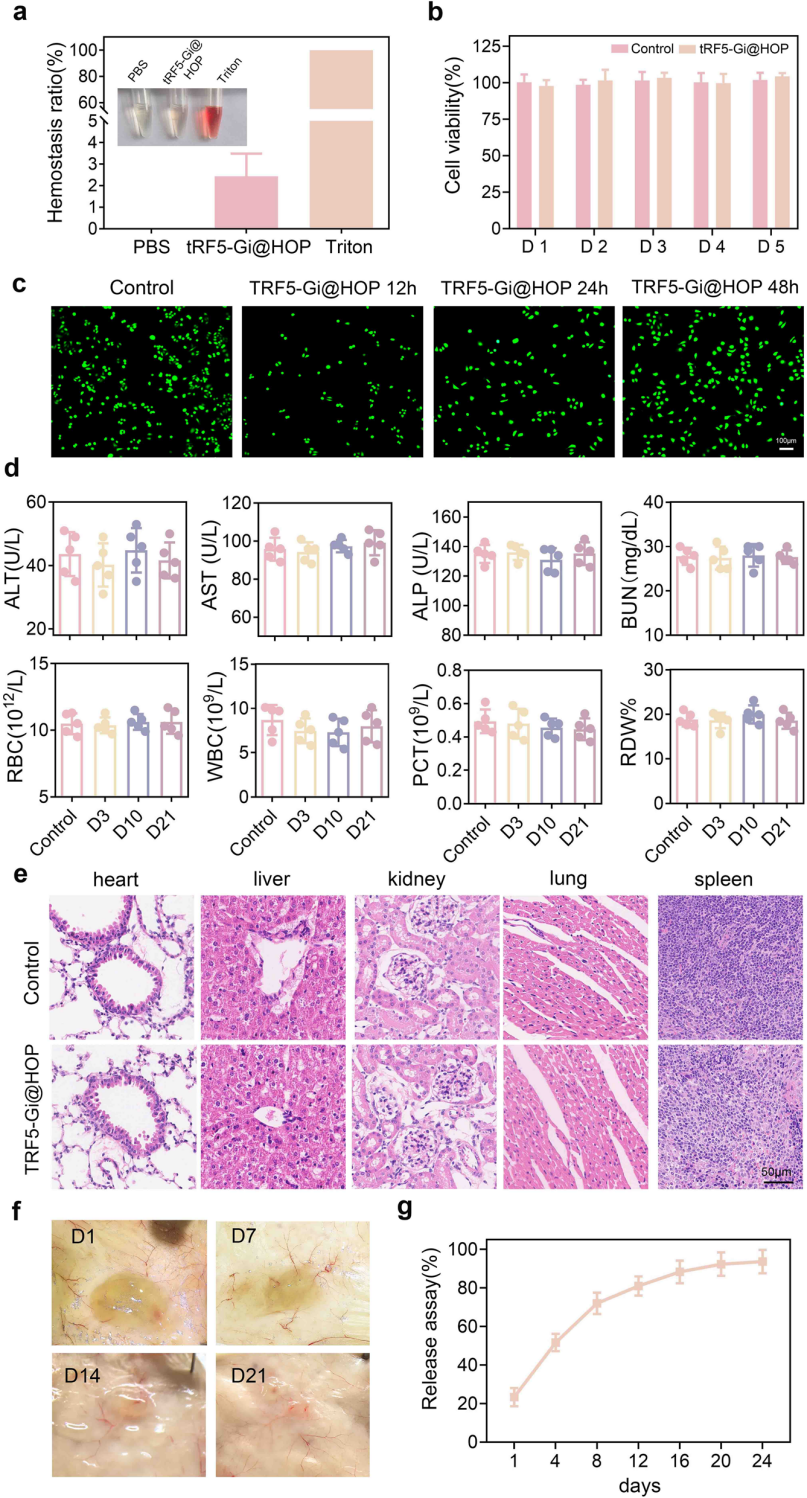
Biocompatibility, biodegradation and drug release behavior of tRF5-Gi@HOP powder. **a** Hemolysis experiments of PBS, Triton, and tRF5-Gi@HOP powder-derived hydrogel. **b** CCK8 assays of THLE-2 cells treated with PBS or tRF5-Gi@HOP powder-derived hydrogel. n = 3. **c** Live/dead staining of THLE-2 cells in coculture with tRF5-Gi@HOP powder-derived hydrogel versus untreated cells. n = 3. **d** Blood parameters for liver and kidney functionality, and blood cell parameters after tRF5-Gi@HOP powder treatment. **e** HE staining of tissue sections of liver, kidney, heart, spleen, and lung collected from mice of control group and tRF5-Gi@HOP powder treatment group. n = 5. **f** The general observation of tRF5-Gi@HOP powder-derived hydrogel after being subcutaneously inserted into dorsal region of mice. **g** The tRF5-Gi release curve of tRF5-Gi@HOP powder-derived hydrogel in vitro. Data shown as means ± SD. P values of i, k and o were calculated using a One-way repeated measures ANOVA test

To evaluate the biocompatibility of tRF5-Gi@HOP powder, a hemolysis experiment was performed. As Fig. 8a shows, the solution became significantly red in the Triton group due to blood cell lysis, whereas it remained nearly bright in the hydrogel and PBS group with hemolysis ratios below 5%. These results suggest that the tRF5-Gi@HOP powder has outstanding hemocompatibility. We then implanted THLE-2 cells in 24-well plates coated with or without the tRF5-Gi@HOP powder-derived hydrogel to evaluate the cytocompatibility of the powder. Figure 8b, c show that no statistical differences were observed between the two groups in terms of cell viability, thus revealing the excellent cytocompatibility of tRF5-Gi@HOP powder. To assess the biocompatibility of tRF5-Gi@HOP powder in vivo, we sprayed it onto the hepatectomy margin of mice and simultaneously regarded liver resection alone as the control group. Blood samples were collected at indicated time points and subjected to biochemical examinations. As Fig. 8d shows, tRF5-Gi@HOP powder treatment did not dramatically alter the serum parameters reflecting liver or kidney function, indicating that it had no obvious effect on these functions. Moreover, the red and white blood cell counts, platelet crit, and red cell distribution width in the mouse blood were not altered following tRF5-Gi@HOP powder treatment, indicating that the powder was friendly to the hematologic system. Consistently, the histopathological examination of multiple mouse organs showed that tRF5-Gi@HOP powder treatment caused no prominent damage to these critical organs (Fig. 8e). These data demonstrate that this powder has good biocompatibility. To assess the biodegradability of tRF5-Gi@HOP powder in vivo, we subcutaneously implanted the derived hydrogel into mice and observed the remaining hydrogel at the indicated time points. Figure 8f shows that the hydrogel bulk shrank in a gradual manner, and only a subtle residue was observed on Day 21, suggesting that the tRF5-Gi@HOP powder had proper biodegradability in vivo. To assess the release dynamics of tRF5-Gi, we immersed the hydrogel in PBS (pH 7.4) and collected the supernatants at predetermined intervals. The concentrations of the released tRF5-Gi in the supernatants were measured through a Nanodrop spectrometer (Thermo Fisher Scientific) for calculating the cumulative release rate of tRF5-Gi. As Fig. 8g shows, the hydrogel released tRF5-Gi gradually with a 93.6% cumulative release rate 24 d after immersion, indicating that the tRF5-Gi@HOP powder has great potential as a delivery platform for tRF5-Gi.

### tRF5-Gi@HOP powder sensitizes RT to enhance NK cell cytotoxicity thereby preventing HCC recurrence

**Fig. 9:**
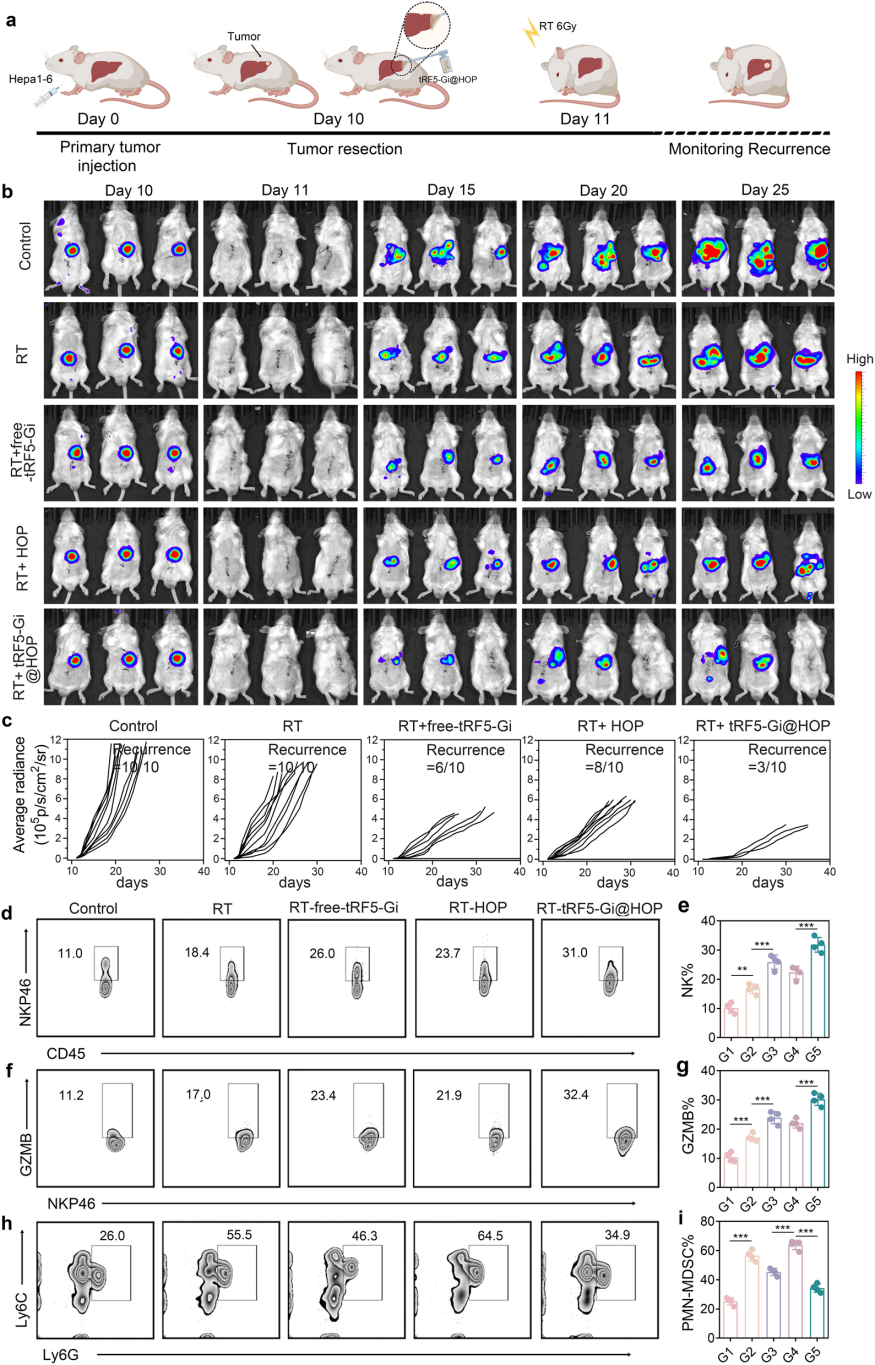
tRF5-Gi@HOP powder sensitizes RT to enhance NK cell cytotoxicity thereby preventing HCC recurrence. **a** Schematic of RT plus tRF5-Gi@HOP powder treatment in orthotopic mouse HCC recurrence model. **b** Dynamic observation of recurrent tumor growth in orthotopic HCC mouse model through bioluminescence imaging. **c** The rates of HCC recurrence in mice of diverse groups. n = 10. **d**, **e** Intratumoral percentage of CD45^+^NKP46^+^ NK cells. **f**, **g** Intratumoral percentage of NKP46^+^ GZMB^+^ NK cells. **h**, **i** Intratumoral percentage of Ly6G^+^ Ly6C ^+^PMN-MDSCs. Data shown as means ± SD. P values of e, g and i were calculated using a One-way repeated measures ANOVA test

To explore whether tRF5-Gi@HOP powder boosts RT-elicited NK cell immunity to combat HCC recurrence post-resection, we first established orthotopic HCC models via hepatic subcapsular injection of Hepa1-6 cells in 50 BALB/c mice (Fig. 9a). These mice underwent hepatectomy to remove tumor masses and were then randomly allocated into five groups: group 1 received no treatment (control), group 2 received single RT (RT), group 3 received RT plus intravenous tRF5-Gi injection (RT-free-tRF5-Gi), group 4 received RT plus surgical margin-localized application of HOP powder without tRF5-Gi (RT-HOP), and group 5 received RT plus tRF5-Gi@HOP powder (RT-tRF5-Gi@HOP). Tumor recurrence was dynamically monitored using fluorescence imaging. As Fig. 9b, c show, a single RT only suppressed recurrent tumor growth to an extent but failed to lower its incidence. However, RT combined with HOP powder, tRF5-Gi injection, or tRF5-Gi@HOP powder not only inhibited recurrent HCC growth but also reduced its occurrence. These combination therapies also significantly improved survival outcomes without significantly affecting the body weight of the mice (Fig. S10). Remarkably, combined treatment with RT and tRF5-Gi@HOP powder performed best in these aspects. Our data clearly indicate that the tRF5-Gi@HOP powder can sensitize RT to combat postoperative HCC recurrence. To ascertain the key mechanism underlying this effect, we first used flow cytometry to analyze the frequency of NKP46^+^ and NKP46^+^ GZMB^+^ NK cells in recurrent HCC tissues collected from mice after 7 d of treatment. Figures 9d–g show that a much higher proportion of NK cell infiltration and function were present in the RT-tRF5-Gi@HOP powder group than in the other groups. These results imply that the synergistic effect of tRF5-Gi@HOP powder and RT in preventing postsurgical HCC recurrence may be attributable to the reinvigoration of NK cell immunity. As previously described, tRF5-GlyGCC can promote Hepa1-6 cells of ITGBL1 and S100A9, which directly and indirectly inhibit NK cell cytotoxicity, respectively. In accordance with these results, tRF5-Gi@HOP powder treatment substantially reversed the RT-induced upregulation of ITGBL1 and S100A9 in HCC tissues (Fig. S11). In addition, our data revealed that the tRF5-GlyGCC/S100A9 signaling axis could impair NK cell antitumor immunity by recruiting PMN-MDSCs to HCC. Accordingly, we explored the effect of tRF5-Gi@HOP powder treatment on the tumor infiltration of PMN-MDSCs in HCC tissues. As Fig. 9h–i show, the proportion of PMN-MDSCs in HCC tissues was markedly elevated after RT, whereas tRF5-Gi@HOP powder treatment substantially reversed the RT-induced tumor accumulation of PMN-MDSCs. Altogether, these results indicate that the tRF5-Gi@HOP powder can reduce the RT-induced secretion of ITGBL1 and S100A9 from tumor cells to reinstate NK cell cytotoxicity, thereby synergizing with RT to prevent HCC recurrence.

### Postoperative anti-adhesion performance of tRF5-Gi@HOP powder

**Fig. 10:**
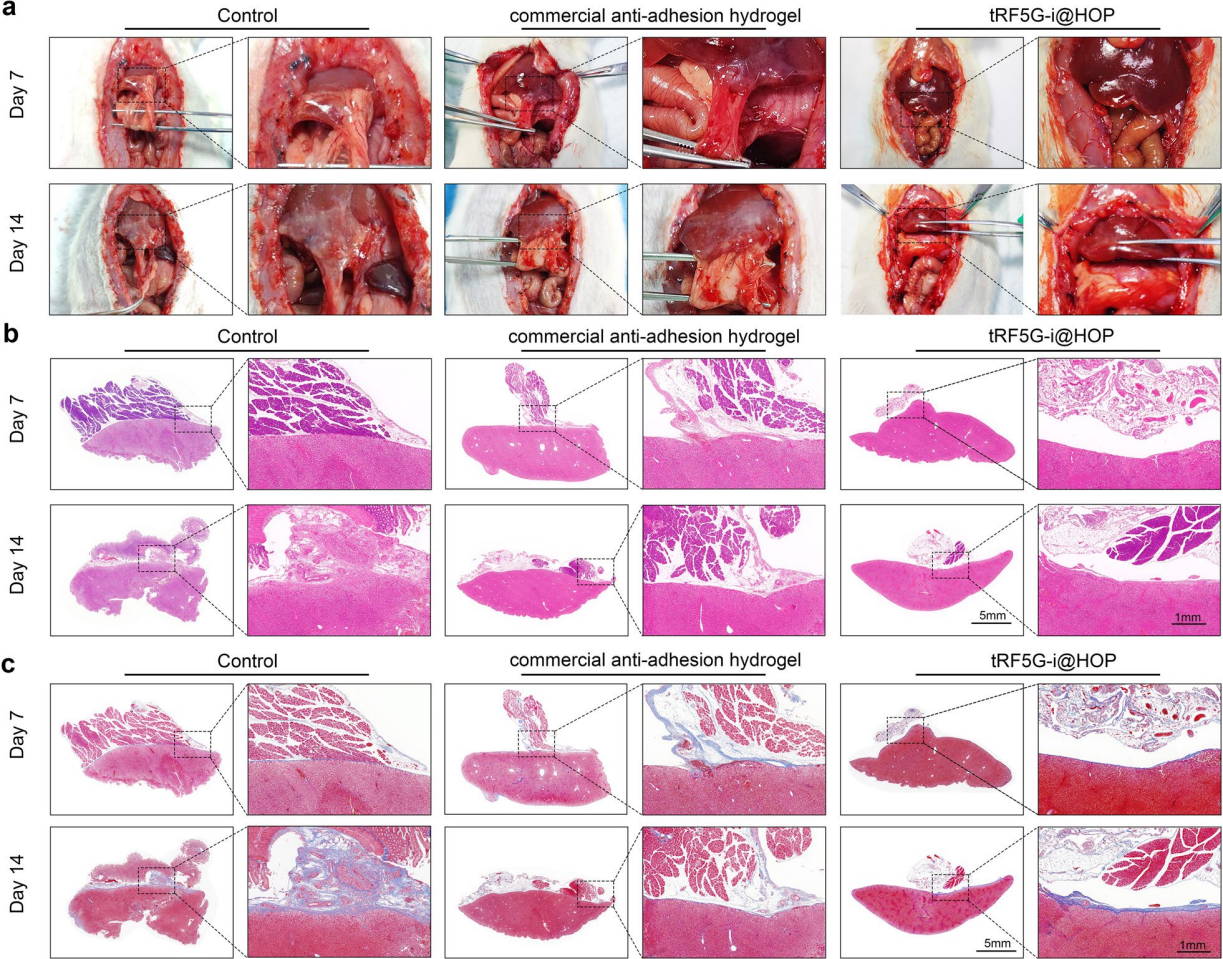
The antiadhesive performance of tRF5-Gi@HOP powder. **a** Adhesion status of rat hepatectomy-induced adhesion models in different groups. **b** H&E staining of adhesive tissues in different groups. **c** Masson’s trichrome staining of adhesive tissues in different groups, n = 3

Postoperative adhesion is another common morbidity in abdominal surgery, which may lead to lifelong risks, such as chronic pain, intestinal obstruction, and even intestinal perforation [39]. Although surgical adhesiolysis can temporarily remove the formed adhesions, it fails to prevent the recurrence of adhesions. In clinical practice, several commercial anti-adhesion biomaterials are used in the form of solid films or powders that can form a solution-like barrier on the surface of injured tissues. However, these biomaterials often provide limited clinical efficacy because they have weak tissue attachment capacity and undergo rapid biodegradation with poor in situ retention in vivo. In recent years, many innovative biomaterials for preventing postoperative adhesions have been developed in preclinical studies, including hydrogel films with Janus adhesion, sprayable dynamic hydrogels, and fast-Janus-gelation powders [40]. Among these newly developed materials, fast-Janus self-gelation powders have attracted much attention due to their several advantages, including delivery convenience, asymmetric tissue adhesion, and excellent adaptability to the dynamic movement of organs [5, 41, 42]. As we demonstrated previously, once deposited onto wet tissues, the lower layer of tRF5-Gi@HOP powder can absorb water at the interface to remove the moisture barriers, during which time the aldehyde groups in oxidized starch react with the amine groups in PAAm to induce the formation of Schiff base bonds to rapidly form a hydrogel in situ. Simultaneously, the aldehyde groups in oxidized starch can interact with the amine groups on wet tissues to yield steady bonding, consequently mediating strong adhesion. Similarly, when the upper powder is hydrated, the aldehyde groups in oxidized starch also react with the amine groups in PAAm to form a hydrogel, which may leave insufficient aldehyde groups to react with the amine groups in wet tissues, preventing the tissues from adhering to the upper surface of the hydrogel and forming a physical barrier between neighboring injured tissues. Considering these findings, we further explored whether tRF5-Gi@HOP powder had anti-adhesion performance. The adhesion of this powder to trimmed pork skin before and after hydration was tested. Movie S3 shows that the pork skin tightly adhered to the unhydrated powder, whereas it failed to adhere to the hydrated powder, indicating the excellent in vitro Janus-adhesion capacity of the powder. Based on this result, we further assessed the in vivo anti-adhesion effect of the tRF5-Gi@HOP powder using a rat hepatectomy-induced adhesion model with reference to a previously reported protocol [5]. In this experiment, a biopolysaccharide-flushing gum solution (a common commercial anti-adhesion biomaterial) or tRF5-Gi@HOP powder was sprayed onto the injured liver area after hepatectomy. No treatment was used as the control group. Once the tRF5-Gi@HOP powder was deposited onto the injured liver area of the rats, PBS was sprayed onto the upper surface of the powder. After two weeks of treatment, the rats were subjected to a double-blind assessment of the degree of abdominal adhesions. As Fig. 10a shows, there were severe adhesions between the liver and the abdominal wall in the control group. Notably, both the biopolysaccharide-flushing gum solution and tRF5-Gi@HOP powder treatments mitigated the degree of liver adhesion, but the latter was superior to the former in this regard. Consistently, the adhesion score of the tRF5-Gi@HOP powder group was significantly lower than that of the biopolysaccharide-flushing gum solution and control groups (Fig. S12). In addition, H&E staining revealed that the liver tissues in the control and biopolysaccharide-flushing gum solution groups significantly adhered to the abdominal wall (Fig. 10b). Moreover, Masson’s trichrome (Masson) staining showed extensive collagen deposition at the adhesion sites of the control and biopolysaccharide-flushing gum solution groups (Fig. 10c). Collectively, these data demonstrate that the tRF5-Gi@HOP powder has excellent anti-adhesion capacity.

## Supplementary Information

Additional file 1.

Additional file 2.

Additional file 3.

Additional file 4.

Additional file 5.

## Data Availability

No datasets were generated or analysed during the current study.
