# Supplementary material for: Radiotherapy plus a self-gelation powder encapsulating tRF5-GlyGCC inhibitor potentiates natural kill cell immunity to prevent hepatocellular carcinoma recurrence
Source: J Nanobiotechnology. 2025 Feb 10;23:100. doi: 10.1186/s12951-025-03133-3 (PMC11809039; doi:10.1186/s12951-025-03133-3)
Supplement: Supplementary file 4 — Additional file 4. [file 12951_2025_3133_MOESM4_ESM.docx]

**Figure S1**


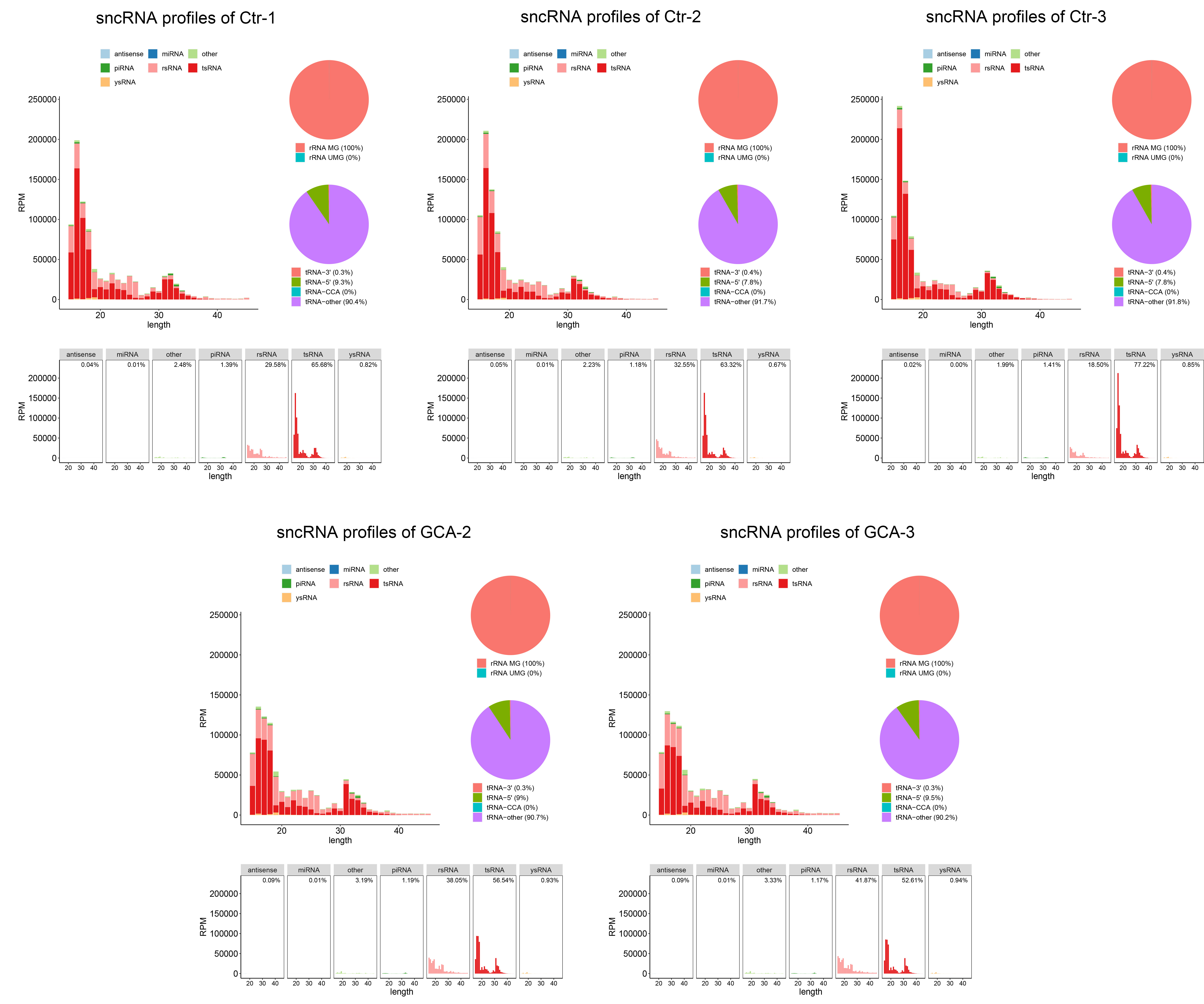


**Figure S1**. The abundance of sncRNAs differently expressed in control Hepa1-6 cells and GCA-treated cells.

**Figure S2**


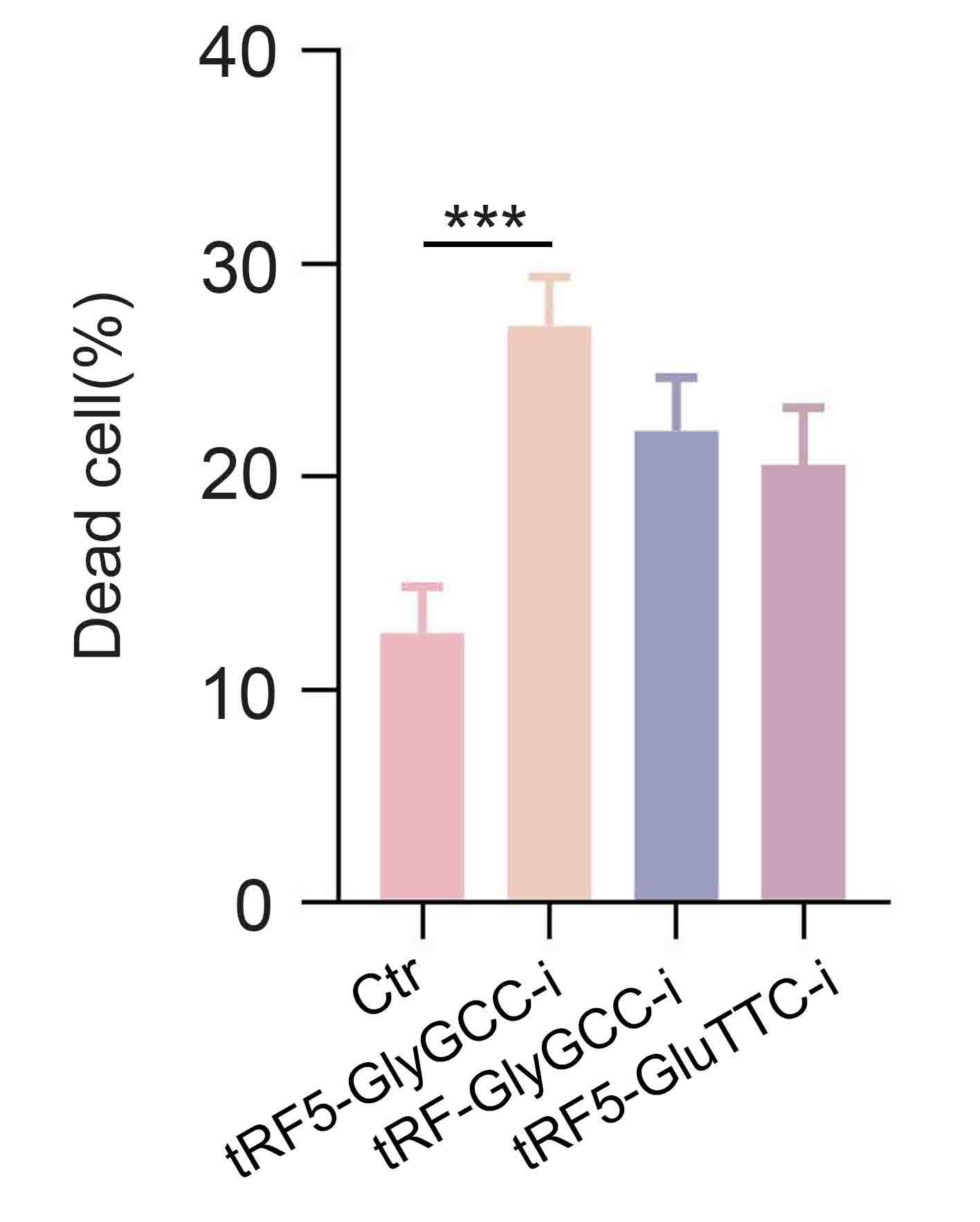


**Figure S2.** Calcein AM staining assay for evaluating the effects of tRF5-G1yTCC, tRF5-GluTCC, tRF-GlyGCC, and tRF-GlyTCC on Hepa1-6 cell sensitivity to NK cell cytotoxicity. n=3. Data shown as means ± SD. P values of a was calculated using a two-sided unpaired Student’s t test.

**Figure S3**


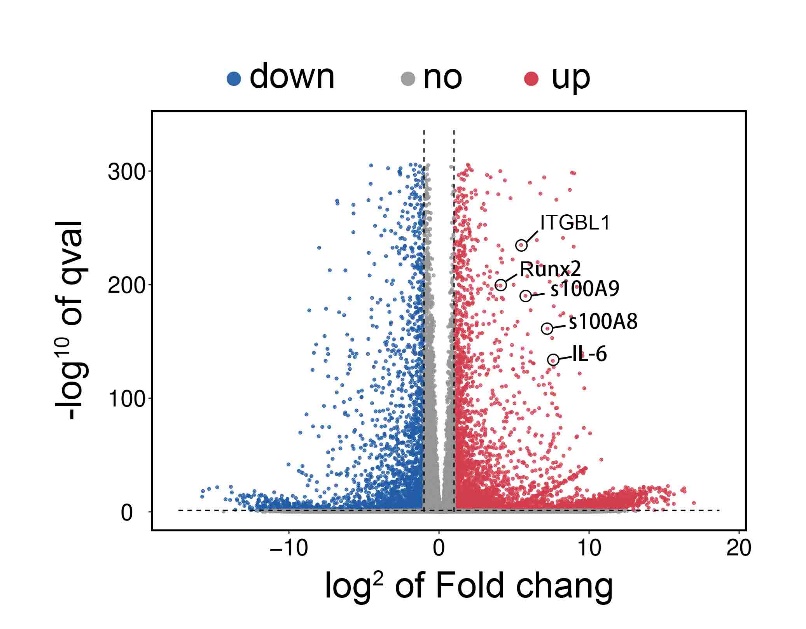


**Figure S3.** The comparison of mRNA profiles in GCA-treated Heapa1-6 cells and control cells through high-throughput sequencing. n=3.

**Figure S4**


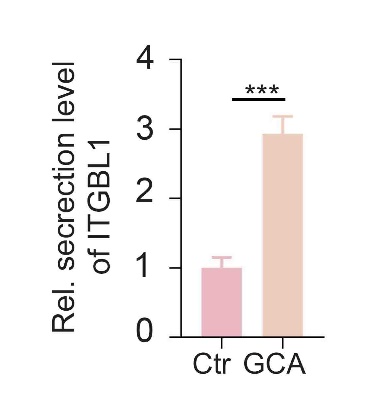


**Figure S4.** Elisa analysis of GCA-meditated effect on ITGBL1 expression in Heapa1-6 cells. n=3. Data shown as means ± SD. P values of a was calculated using a two-sided unpaired Student’s t test.

**Figure S5**


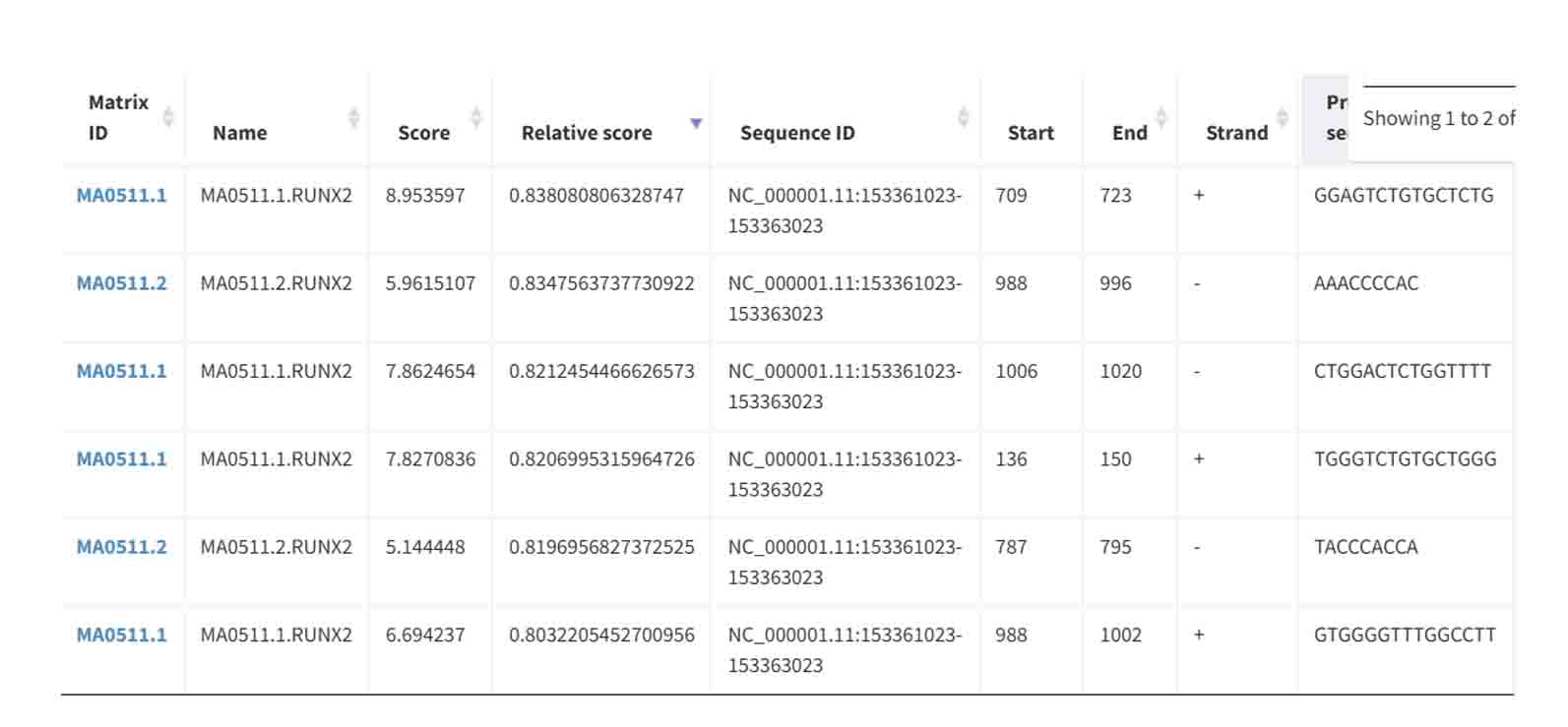


**Figure S5.** JAS-PAR database analysis predicted putative Runx2-binding sites in the S100A9 promoter.

**Figure S6**


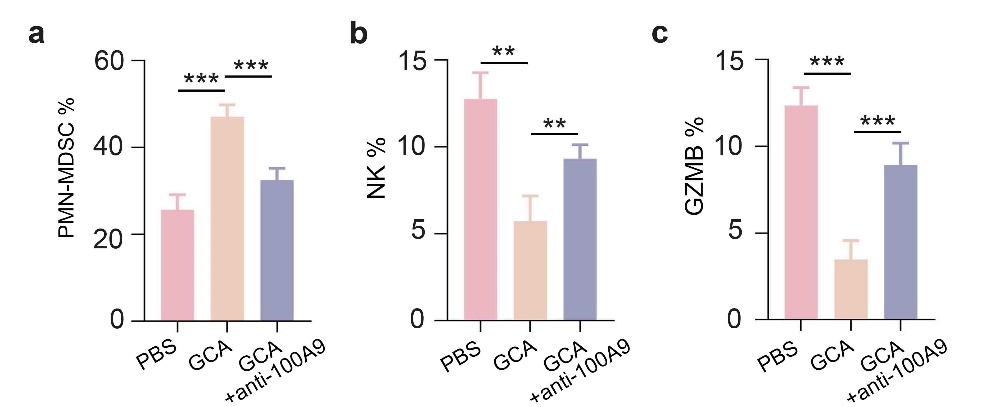


**Figure S6.** a: Intratumoral percentage of Ly6G^+^ Ly6C ^+^PMN-MDSCs. b: Intratumoral percentage of CD45^+^NKP46^+^ NK cells. c: Intratumoral percentage of NKP46^+^ GZMB^+^ NK cells. n=3. Data shown as means ± SD. P values were calculated using a One-way repeated measures ANOVA test.

**Figure S7**


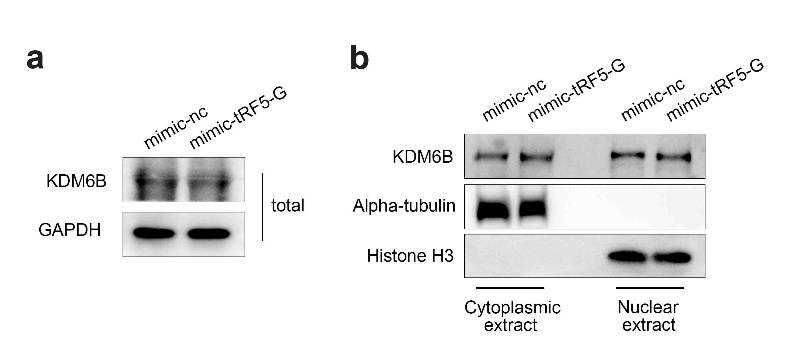


**Figure S7.** a: The effect of tRF5-GlyGCC mimics on the total protein expression of KDM6B. b.The effect of tRF5-GlyGCC mimics on the expressions of nuclear and cytoplasmic KDM6B protein.

**Figure S8**


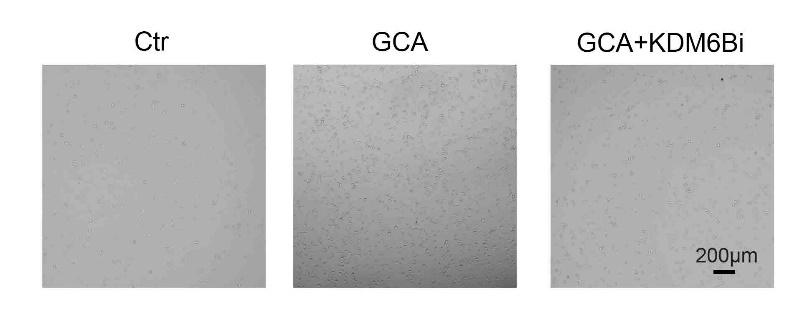


**Figure S8.** The capacities of hepa1-6 cells in control, GCA, and GCA plus KDM6B inhibitor to attract PMN-MDSCs in vitro.

**Figure S9**


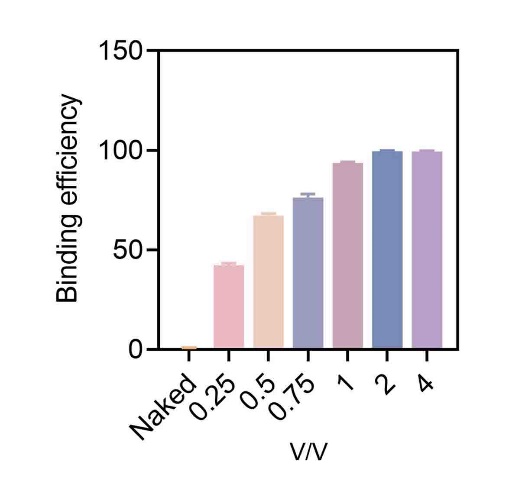


**Figure S9.** The binding efficiency of NMOFs to tRF5-Gi at different volume ratios.

**Figure S10**

**Figure S10.** a: Survival prognosis of mice in different treatment groups. n=10. b: Dynamic weights of mice in different groups. n=10.


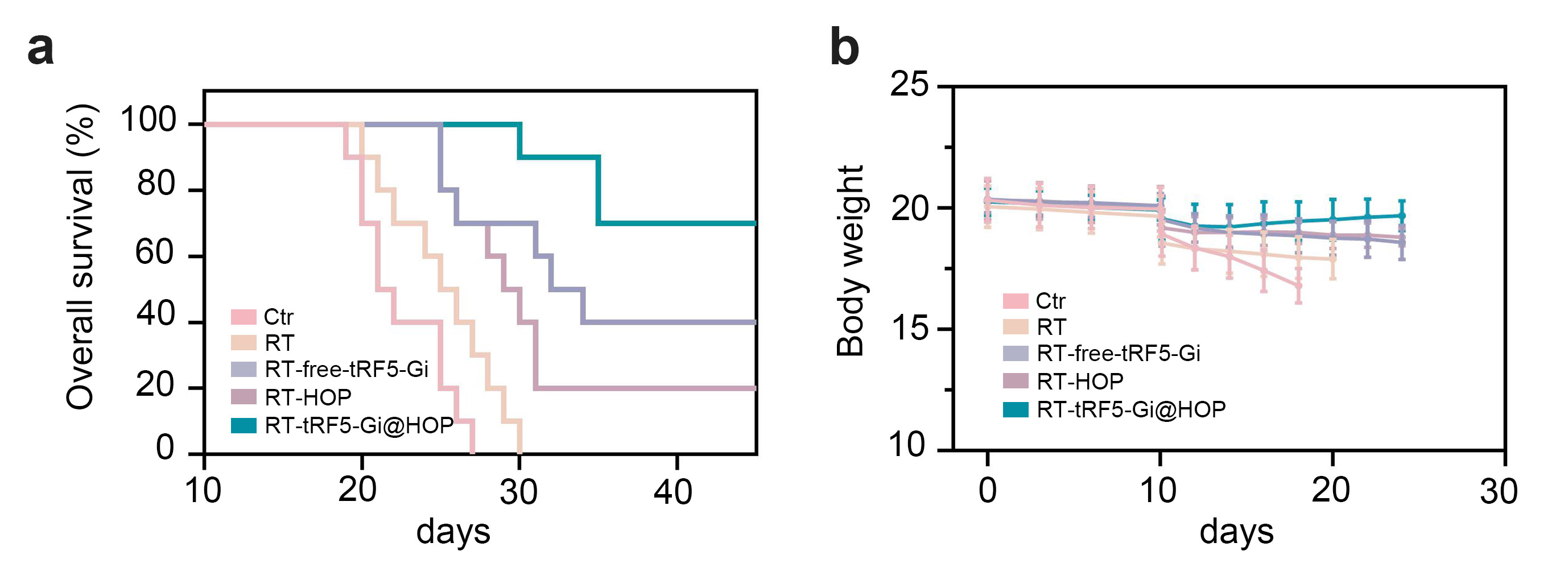


**Figure S11**


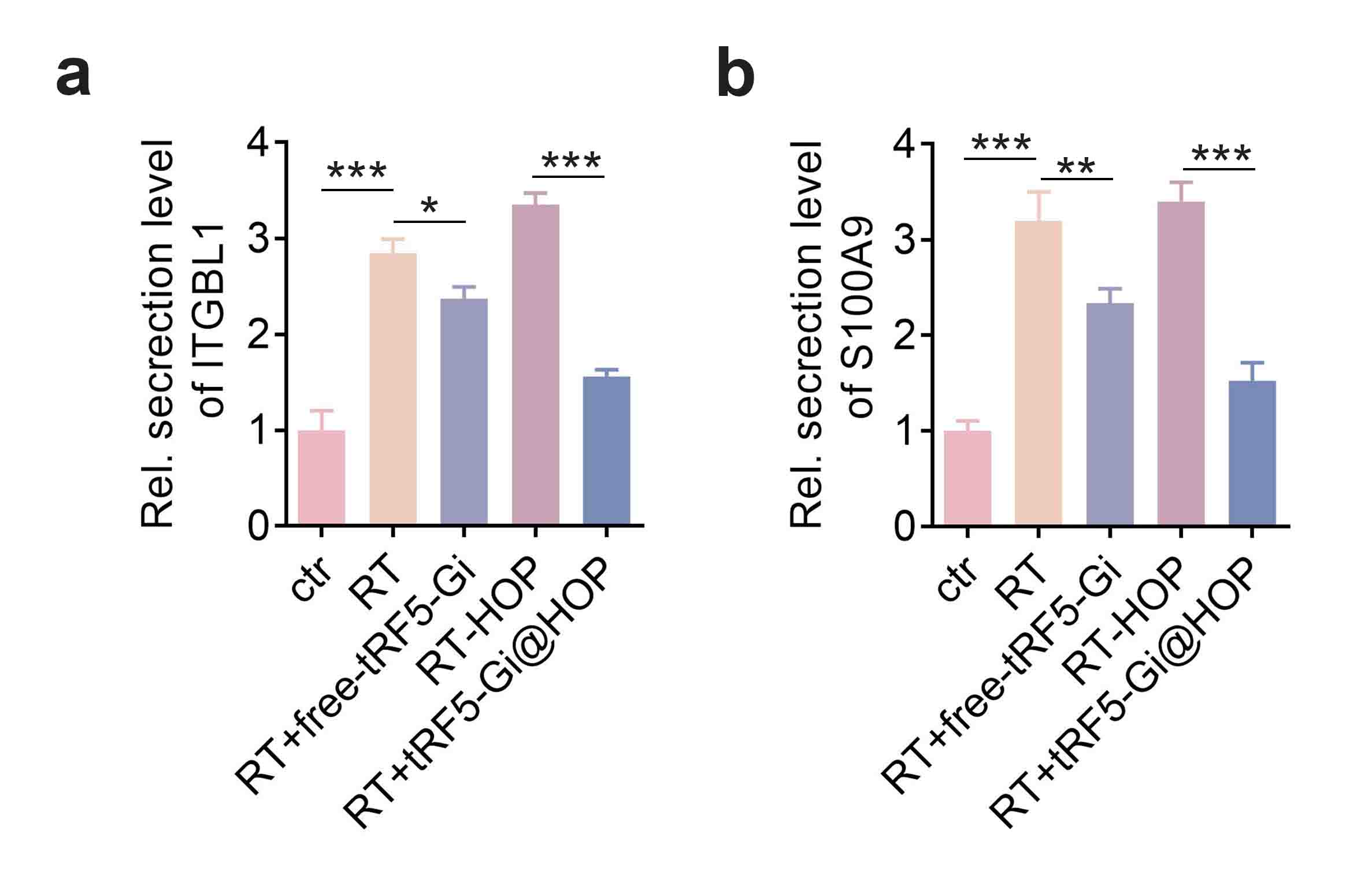


**Figure S11.** a: ELISA analysis of ITGBL1 and S100A9 secretion from tumor tissue of distinct groups. n=4. Data shown as means ± SD. P values were calculated using a One-way repeated measures ANOVA test.

**Figure S12**


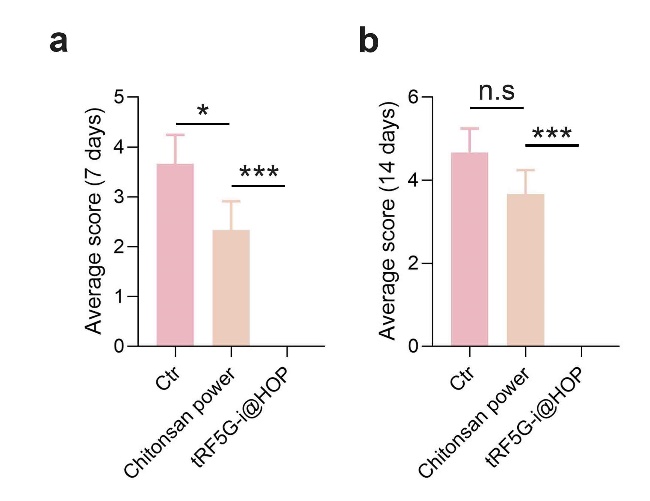


**Figure S12. a**: Average adhesion scores of rats in control, Chitonsan, and tRF5@HOP powder treatment groups on day 7. **b**: Average adhesion scores of rats in control, Chitonsan, and tRF5@HOP powder treatment groups on day 14. n=3. Data shown as means ± SD. P values were calculated using a One-way repeated measures ANOVA test.
