# Supplementary material for: Radiotherapy plus a self-gelation powder encapsulating tRF5-GlyGCC inhibitor potentiates natural kill cell immunity to prevent hepatocellular carcinoma recurrence
Source: J Nanobiotechnology. 2025 Feb 10;23:100. doi: 10.1186/s12951-025-03133-3 (PMC11809039; doi:10.1186/s12951-025-03133-3)
Supplement: Supplementary file 5 — Additional file 5. [file 12951_2025_3133_MOESM5_ESM.docx]

| **name** | **sequence** |
| --- | --- |
| tRNF-Gly-GCC_5_end | GCATGGGTGGTTCAGTGGTAGAATTCTCGCCTG |
| tRNF-Glu-TTC_5_end | TCCCACATGGTCTAGCGGTTAGGATTCCTGGTTTTC |
| tRNF-Gly-GCC | ATGGGTGGTTCAGTGGTAGAATTCTCGCCTGC |
| tRNF-Gly-TCC | GTGGTGAGCATAGCTGCCTTC |
| Runx2 forward | GACTGTGGTTACCGTCATGGC |
| Runx2 reverse | ACTTGGTTTTTCATAACAGCGGA |
| ITGBL1 forward | GCAGAGTCCGAACGCAGAT |
| ITGBL1 reverse | ACACAGTGGACCGAAGTAGGT |
| S100A9 forward | ATACTCTAGGAAGGAAGGACACC |
| S100A9 reverse | TCCATGATGTCATTTATGAGGGC |
| S100A8 forward | AAATCACCATGCCCTCTACAAG |
| S100A8 reverse | CCCACTTTTATCACCATCGCAA |
| IL-6 forward | TAGTCCTTCCTACCCCAATTTCC |
| IL-6 reverse | TTGGTCCTTAGCCACTCCTTC |
| GAPDH forward | AGGTCGGTGTGAACGGATTTG |
| GAPDH reverse | TGTAGACCATGTAGTTGAGGTCA |

**Table S1**
